# An integrated gene-to-outcome multimodal database for metabolic dysfunction-associated steatotic liver disease

**DOI:** 10.1038/s41591-023-02602-2

**Published:** 2023-10-30

**Authors:** Timothy J. Kendall, Maria Jimenez-Ramos, Frances Turner, Prakash Ramachandran, Jessica Minnier, Michael D. McColgan, Masood Alam, Harriet Ellis, Donald R. Dunbar, Gabriele Kohnen, Prakash Konanahalli, Karin A. Oien, Lucia Bandiera, Filippo Menolascina, Anna Juncker-Jensen, Douglas Alexander, Charlie Mayor, Indra Neil Guha, Jonathan A. Fallowfield

**Affiliations:** 1 Institute for Regeneration and Repair, University of Edinburgh, Edinburgh, UK; 2 Edinburgh Pathology, University of Edinburgh, Edinburgh, UK; 3 Edinburgh Genomics (Bioinformatics), University of Edinburgh, Edinburgh, UK; 4 OHSU-PSU School of Public Health, Oregon Health & Sciences University, Portland, OR USA; 5 Knight Cancer Institute Biostatistics Shared Resource, Oregon Health & Sciences University, Portland, OR USA; 6 Precision Medicine Scotland-Innovation Centre (PMS-IC), University of Glasgow, Glasgow, UK; 7 Pathology Department, Queen Elizabeth University Hospital, Glasgow, UK; 8 School of Engineering, Institute of Bioengineering, University of Edinburgh, Edinburgh, UK; 9 Centre for Engineering Biology, University of Edinburgh, Edinburgh, UK; 10 NeoGenomics Laboratories, Fort Myers, FL USA; 11 NHS Greater Glasgow and Clyde Safe Haven, Glasgow, UK; 12 National Institute of Health Research (NIHR) Nottingham Biomedical Research Centre, Nottingham University Hospitals NHS Trust and University of Nottingham, Nottingham, UK

**Keywords:** Translational research, Data integration, Genetic databases

## Abstract

Metabolic dysfunction-associated steatotic liver disease (MASLD) is the commonest cause of chronic liver disease worldwide and represents an unmet precision medicine challenge. We established a retrospective national cohort of 940 histologically defined patients (55.4% men, 44.6% women; median body mass index 31.3; 32% with type 2 diabetes) covering the complete MASLD severity spectrum, and created a secure, searchable, open resource (SteatoSITE). In 668 cases and 39 controls, we generated hepatic bulk RNA sequencing data and performed differential gene expression and pathway analysis, including exploration of gender-specific differences. A web-based gene browser was also developed. We integrated histopathological assessments, transcriptomic data and 5.67 million days of time-stamped longitudinal electronic health record data to define disease-stage-specific gene expression signatures, pathogenic hepatic cell subpopulations and master regulator networks associated with adverse outcomes in MASLD. We constructed a 15-gene transcriptional risk score to predict future hepatic decompensation events (area under the receiver operating characteristic curve 0.86, 0.81 and 0.83 for 1-, 3- and 5-year risk, respectively). Additionally, thyroid hormone receptor beta regulon activity was identified as a critical suppressor of disease progression. SteatoSITE supports rational biomarker and drug development and facilitates precision medicine approaches for patients with MASLD.

## Abstract (web summary)

SteatoSITE is an open resource that integrates histopathologic assessments, transcriptomic data and longitudinal electronic health records for a cohort of 940 patients with metabolic dysfunction-associated steatotic liver disease.

## Main

Metabolic dysfunction-associated steatotic liver disease (MASLD), previously termed non-alcoholic fatty liver disease (NAFLD)^1^, is a growing and underappreciated global public health concern affecting more than one in four adults (over two billion people) and imposing a substantial burden of ill health and socioeconomic problems^2,3^. It is characterized by the presence of cardiometabolic risk factors such as obesity, type 2 diabetes, hypertension and dyslipidemia and is associated with increased morbidity and mortality from cardiovascular disease, chronic kidney disease and many cancers. The histopathological spectrum of MASLD includes isolated fatty liver (simple steatosis) and metabolic dysfunction-associated steatohepatitis (MASH), previously termed non-alcoholic steatohepatitis (NASH), the inflammatory subphenotype of MASLD that can lead to progressive liver fibrosis, cirrhosis and hepatocellular carcinoma (HCC). Notably, over the past ten years, there has been a tenfold increase in the number of patients with MASLD-related cirrhosis who require a liver transplant^4^, and Bayesian modeling forecasts a staggering global prevalence rate of 55.7% by 2040 (ref. ^5^).

The pathogenesis of MASLD is complex and multifactorial^6,7^. The primary insult of excess lipid accumulation (‘substrate overload’) is followed by variable contributions from pathogenic drivers including lipotoxicity and immune-mediated inflammation, and disease progression is further enhanced or suppressed by modifiers such as genetic variants^8^ and gut microbiota dysbiosis^9,10^. Crucially, not all people with a fatty liver go on to develop adverse liver-related events or die^11^, but there is a critical lack of prognostic biomarkers to enable innovations in clinical care pathways and personalized approaches in MASLD. The importance of histological fibrosis in predicting outcomes in MASLD has been highlighted by several studies^11–13^, with discrete fibrosis stages dramatically influencing the risk of all-cause mortality and liver-related morbidity and mortality. Yet fibrosis progression rates and outcome prediction in patients remain uncertain, and clinical trials that have been used post hoc to understand the natural history of MASLD are limited by selection bias and rigid entry criteria^14^.

There are no licensed pharmacotherapies for MASLD. Numerous drugs with plausible biological targets have failed to show robust efficacy in late-phase interventional trials, especially in patients with cirrhosis^15^. Consequently, the field is shifting focus to combination drug regimens, and, increasingly, researchers are leveraging patient-centered big data to bolster drug discovery efforts^16^. However, the most efficacious drugs and therapeutic combinations to mitigate adverse clinical outcomes in MASLD remain undefined.

To address these issues and provide a resource suitable for the development of precision medicine strategies in MASLD, we established SteatoSITE: to our knowledge, the world’s first data commons^17^ for MASLD research. SteatoSITE integrates multimodal multiscale retrospective data at a national level from 940 cases across Scotland, consisting of quantitative histopathological assessment of archival liver tissues, hepatic bulk RNA sequencing (RNA-seq) and routine clinical data extracted from electronic health records (EHRs) and other administrative sources. Distinctively, SteatoSITE is outcome-rich, with data curated in a longitudinal time-stamped format comprising 5.67 million days of clinical follow-up, enabling translational analyses to elucidate the pathogenesis of MASLD and accelerating the discovery of potential prognostic biomarkers and tractable therapeutic targets. Here, we describe and use this unique resource, providing a comprehensive analysis of hepatic gene expression and cell-type composition across the complete MASLD spectrum by identifying transcription-factor-regulated gene networks (regulons) associated with disease progression and developing a novel transcriptome-based risk-prediction model for hepatic decompensation.

## Results

### Cohort composition

**Fig. 1:**
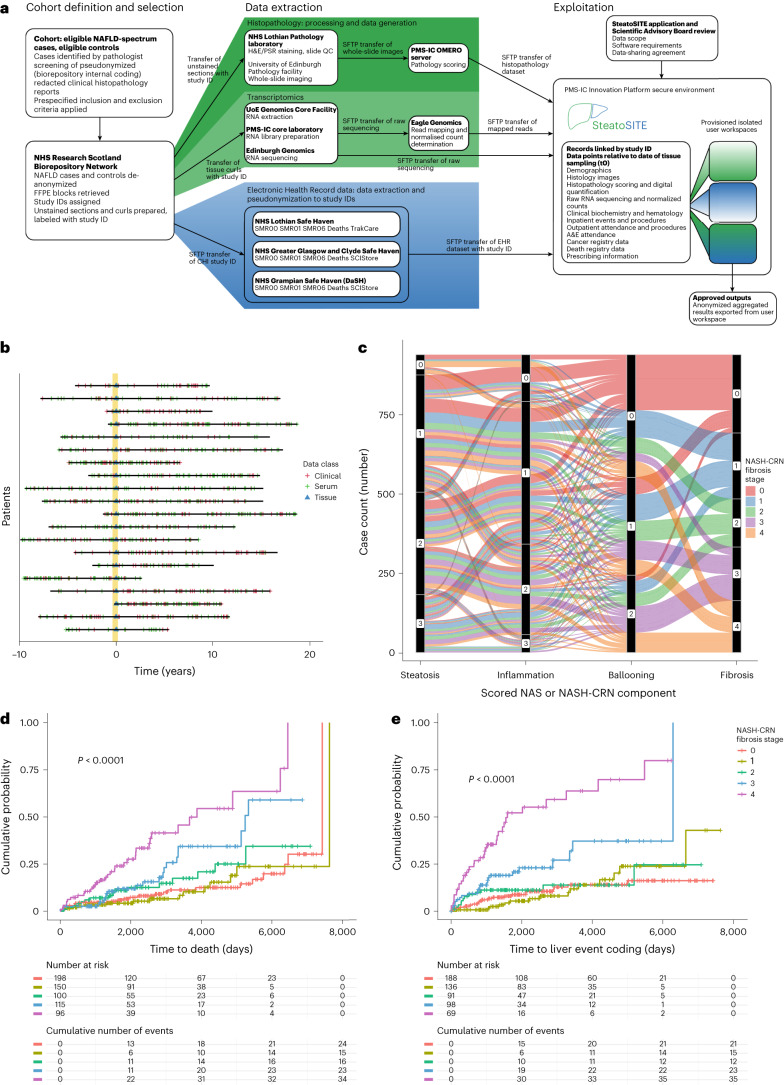
Clinicopathological correlations. **a**, SteatoSITE Data Commons overview. **b**, Schematic diagram in which horizontal lines are individual patient timelines decorated with a variable amount of multimodal data preceding or following the date of liver tissue sampling (time zero, represented by the vertical yellow line). **c**, Alluvial diagram illustrating the relationship between the scored histopathological features of liver samples. **d**,**e**, Kaplan–Meier time-to-event analysis with log-rank test *P* value for all-cause mortality (*P* < 0.0001) (**d**) and hepatic decompensation events (*P* < 0.0001) (**e**) in patients with biopsies showing early disease (fibrosis stages F0 to F2), bridging fibrosis (stage F3) and cirrhosis (stage F4). A&E, accident and emergency; SFTP, Secure File Transfer Protocol.

**Extended Data Fig. 1:**
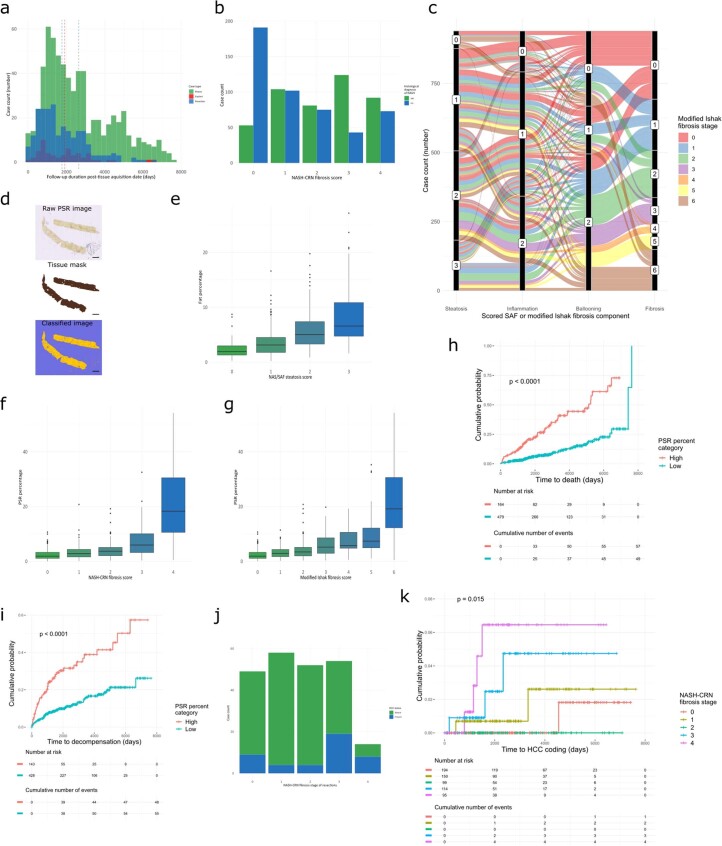
Additional histopathological analyses. **a**, Clinical follow-up depth (in days) according to specimen type. **b**, Clinical assessment of the presence or absence of a histological diagnosis of steatohepatitis versus NASH-CRN stage. **c**, Alluvial diagram illustrating the relationship between the scored SAF and modified Ishak stage features. **d**, A representative whole-slide image of a PSR-stained section with the associated tissue mask and applied classifier showing PSR-positive (red) and fat droplets (purple) from the QuPath quantitative pathology pipeline; only pixels present within the tissue mask are used for PSR and fat quantitation. Pixel classification using the same classifier always returns the same values so was only undertaken once. Scale bar, 2 mm. **e**, The amount of steatosis from image quantification versus NAS steatosis assigned scores from the 922 cases with quantifiable whole-slide images. **f**, The PSR percentage from image quantification versus assigned NASH-CRN scores from the 922 cases with quantifiable whole-slide images. **g**, The PSR percentage from image quantification versus assigned modified Ishak scores from the 922 cases with quantifiable whole-slide images. All boxplots show median (centre line), first and third quartiles (lower and upper box limits), 1.5× interquartile range (whiskers). Computationally-derived scar proportion can be used to stratify the risk of all-cause mortality (**h**) and hepatic decompensation (**i**); log–rank test *P-*values < 0.0001. **j**, Count of presence or absence of hepatocellular carcinoma (HCC) in resection cases versus assigned NASH-CRN stage. **k**, Kaplan–Meier time-to-event analysis for HCC in patients with biopsies showing early disease (fibrosis stages F0 to F2), bridging fibrosis (stage F3) and cirrhosis (stage F4); log–rank test *P*-value = 0.015.

SteatoSITE is a secondary-care, outcome-enriched cohort of 940 cases from the three participating NHS Scotland Biorepositories (Lothian, Greater Glasgow, and Clyde and Grampian) (Fig. 1a), representing the full histological spectrum from normal liver tissue to MASLD-related cirrhosis. Demographic and phenotypic characteristics of the cohort are shown in Table 1. Cases with a liver tissue sample acquired between January 2000 and October 2019 were selected. All patients were ≥18 years of age at the tissue sampling date. Data from EHRs and national datasets were retrieved, where available, from a period between ten years before the tissue sampling date until May 2020, comprising more than 5.67 million days (or roughly 15,547 years) of comprehensive routine clinical data, and annotated patient timelines were created (Fig. 1b and Supplementary Table 1). Further detail of the temporal coverage of blood test results is shown in Supplementary Table 2. Indications for biopsy or resection on the basis of a review of the information submitted with each specimen as part of routine clinical care are shown in Supplementary Table 3. The post-tissue sampling periods for biopsies, resections and explants are shown in Extended Data Fig. 1a.

**Table 1:** Demographics and clinical characteristics of SteatoSITE cohort

	NASH-CRN fibrosis stage				
Variable	0	1	2	3	4
Age, mean (s.d.)					
Years	49.8 (13.9)	53 (14.5)	56.8 (13.6)	59.1 (11.5)	59.9 (9.8)
Gender, number (%)					
Men	137 (55.5)	124 (59.6)	92 (60.5)	85 (50.3)	83 (50.6)
Women	110 (44.5)	84 (40.4)	60 (39.5)	84 (49.7)	81 (49.4)
Ethnicity, number (%)					
White	153 (16.3)	137 (14.6)	90 (9.6)	106 (11.3)	106 (11.3)
Asian	8 (0.9)	6 (0.6)	3 (0.3)	3 (0.3)	2 (0.2)
African	1 (0.1)	0 (0)	0 (0)	0 (0)	0 (0)
Unknown	85 (9)	65 (6.9)	59 (6.3)	60 (6.4)	56 (6)
BMI					
Median (IQR)	30 (7.3)	32.2 (10.3)	31 (5.2)	33.1 (7)	31.7 (7.4)
Unknown BMI, number	175	146	116	120	105
Diabetes, number (%)					
Yes	33 (13.4)	48 (23.1)	42 (27.6)	79 (46.7)	105 (64)
No	214 (86.6)	160 (76.9)	110 (72.4)	90 (53.3)	59 (36)
SIMD quintile, number (%)					
1	21 (2.2)	27 (2.9)	23 (2.4)	29 (3.1)	25 (2.7)
2	19 (2)	30 (3.2)	14 (1.5)	16 (1.7)	26 (2.8)
3	28 (3)	19 (2)	17 (1.8)	21 (2.2)	24 (2.6)
4	23 (2.4)	18 (1.9)	10 (1.1)	14 (1.5)	23 (2.4)
5	43 (4.6)	37 (3.9)	32 (3.4)	36 (3.8)	26 (2.8)
Unknown SIMD	113 (12)	77 (8.2)	56 (6)	53 (5.6)	40 (4.3)
Liver enzymes, median (IQR)					
ALT (U per l)	66 (78)	90 (106.5)	85 (109.5)	77.5 (98.75)	53 (110.25)
AST (U per l)	38 (29)	49 (42)	48 (36.25)	53 (46)	44.5 (37.5)
ALP (U per l)	88 (50.5)	82.5 (43.75)	81 (47)	93.5 (53.5)	107 (69)
Bilirubin (µmol per l)	11 (9)	11 (8.25)	12 (10)	14 (12)	19 (37.5)
Other laboratory results, median (IQR)					
Prothrombin time (s)	12 (2)	12 (2)	12 (2)	13 (1.25)	14 (7)
INR	1 (0.1)	1 (0.1)	1 (0.1)	1.1 (0.2)	1.2 (0.4)
Albumin (g per l)	40 (10)	39 (8)	38 (12)	37 (9)	33.5 (16)
Platelets (×10^9^ per l)	237 (100)	230 (94)	213 (96.5)	198 (104.75)	130.5 (103)
Creatinine (µmol per l)	73 (17)	72 (21)	75 (18.25)	70 (22)	71 (26)
Non-invasive tests, median (IQR)					
FIB-4	1 (1)	1.1 (1.2)	1.4 (1.2)	1.8 (1.8)	2.4 (1.9)
Transient elastography (kPa)	8.2 (5.7)	10.9 (5.5)	12.3 (6.9)	14.4 (9.4)	27.3 (34.3)
Prescription, number (%)					
Statin	19 (7.7)	32 (15.4)	18 (11.8)	27 (16)	32 (19.5)
Non-selective beta blocker	1 (0.4)	1 (0.5)	5 (3.3)	4 (2.4)	7 (4.3)
Specimen type, number (%)					
Biopsy	198 (80.2)	150 (72.1)	100 (65.8)	115 (68)	96 (58.5)
Explant	0 (0)	0 (0)	0 (0)	2 (1.2)	54 (32.9)
Resection	49 (19.8)	58 (27.9)	52 (34.2)	52 (30.8)	14 (8.5)
Total case number	247	208	152	169	164

ALP, alkaline phosphatase; ALT, alanine transaminase; AST, aspartate transaminase; FIB-4, fibrosis 4; INR, international normalized ratio; IQR, interquartile range; s.d., standard deviation; U per l, units per liter; µmol per l, mircomoles per liter.

### Histopathological characterization

Scans of hematoxylin and eosin (H&E)- and picrosirius red (PSR)-stained sections from each case were assessed by one of three consultant pathologists with a specialist interest in liver pathology. From the H&E-stained sections, NAFLD activity scores (NASs), components of the Steatosis, Activity and Fibrosis (SAF) scoring system and a score-independent clinical histological diagnosis of NASH were given. From the PSR-stained sections, a NASH-Clinical Research Network (NASH-CRN) and modified Ishak score for staging of fibrosis were given. The inter-rater agreement levels of the scoring pathologists (Supplementary Table 4), assessed on a set of 20 cases after the scoring-harmonization exercise, were at the upper end of the expected ranges^18,19^. Spearman’s rho correlation coefficient was used to assess the relationship between modified Ishak fibrosis scores and NASH-CRN fibrosis scores, demonstrating the expected strong positive correlation (*r*_s_ = 0.98, *P* < 2.2 × 10^−16^).

Of the 940 cases, 659 were needle biopsies, 225 were from hepatic resections and 56 were explants for clinically end-stage NAFLD cirrhosis. A clinical histological diagnosis of NASH was given in 455 (48.4%) cases (Extended Data Fig. 1b). Roughly equal numbers of cases were scored at each point of the NASH-CRN fibrosis scale. The relationship of the NAS components and the NASH-CRN fibrosis score are shown in Fig. 1c, and those of the SAF system with modified Ishak fibrosis stage are shown in Extended Data Fig. 1c.

A pixel classifier was trained to identify fat and PSR-positive tissue in scans of PSR-stained sections (Extended Data Fig. 1d). All classified images were manually quality-controlled, and any images containing large fragments of liver capsule, large portal tracts or hilar tissue or with an artifact that was easily ignored during subjective assessment but erroneously computationally classified were removed. The derived fat percentage of the tissue correlated with the assigned steatosis score of the NAS (and SAF) systems, as expected (*r*_s_ = 0.53, *P* < 2.2 × 10^−16^; Extended Data Fig. 1e). The derived PSR-positive percentage of the tissue also correlated with both NASH-CRN (*r*_s_ = 0.61, *P* < 2.2 × 10^−16^; Extended Data Fig. 1f) and modified Ishak (*r*_s_ = 0.61, *P* < 2.2 × 10^−16^; Extended Data Fig. 1g) assigned scores.

### Association of histological features with clinical outcomes

The extensive annotation of individual case timelines with clinical data, anchored by the specimen date and retrieved from the EHRs and national datasets, allowed the relationship between data derived from the histological sections and patient outcomes to be examined. Using the 659 needle biopsy cases only, the clear and expected relationship between assigned NASH-CRN fibrosis stage and all-cause mortality was observed (Fig. 1d). Stepwise increases in mortality were evident through progression from stage F0 to F4. An unbiased algorithmic approach to cluster survival curves^20^ created two clusters (F0,1,2) and (F3,4), supporting the hypothesis that fibrous bridging of vascular structures is a critical pathophysiological event with prognostic importance in progressive fibrogenesis.

Standard Cox regression modeling of all-cause mortality using age at biopsy, gender and NASH-CRN fibrosis stage as covariates showed that age and NASH-CRN fibrosis stage F4 were independently associated with higher all-cause mortality (Supplementary Table 5). Median intervals to death and censoring of included cases are shown in Supplementary Table 6. A ‘pathology scoring only’ model was also examined; only fibrosis stage was predictive of survival on multivariate analysis (Supplementary Table 7).

The predictive value of the computationally derived PSR-positive proportion in the biopsy cases where a value was available was also separately examined. Increased PSR content in a biopsy was associated with increased risk of death, hazard ratio 1.06 (95% confidence interval 1.045–1.075, *P* < 2 × 10^−16^). To illustrate the potential value of computational pathology in providing prognostic information about overall survival, maximally selected rank statistics were used to determine the optimal PSR percentage cutpoint that divided samples into high- and low-risk groups; the Kaplan–Meier estimator curves are shown in Extended Data Fig. 1h.

The annotated patient timeline of 183 of the 659 biopsy cases contained an outcome coding for at least one of the events defined by expert consensus to represent decompensation in cirrhotic patients^21^ or, using UK Operations/Procedure coding data, identifying activity relating to cirrhosis-related hospital admissions^22^. This high number of events contrasts with a published prospective observational study^11^. In SteatoSITE, using only the 106 cases for which an event coding related to decompensation was not present on the patient timeline before the biopsy, F3 and F4 NASH-CRN fibrosis stage was predictive of subsequent decompensation (Fig. 1e). Kaplan–Meier estimator curves for liver-related events (excluding death) associated with F0, F1 and F2 were placed in the same cluster by an unbiased clustering approach, but those associated with F3 and F4 were distinct.

Cox regression modeling of hepatic decompensation events on the cause-specific hazard, with death as a competing risk, using age at biopsy, gender and NASH-CRN fibrosis stage, showed that NASH-CRN fibrosis stages F3 and F4 were associated with increased decompensation events (Supplementary Table 8); Fine–Gray regression for the proportional-hazards modeling of the subdistribution hazard is also reported. Median intervals to decompensation or censoring (death or end of follow-up) of included cases are shown in Supplementary Table 9. A ‘pathology scoring only’ model for hepatic decompensation (with death as a competing risk) was also examined; only fibrosis stage was predictive of hepatic decompensation on multivariate analysis (Supplementary Table 10).

The value of the computationally derived PSR-positive proportion in the biopsy cases where a value was available was also separately examined for hepatic decompensation, with death as a competing risk. Increased PSR content in a biopsy was associated with increased risk of hepatic decompensation, hazard ratio 1.07 (95% confidence interval 1.05–1.08, *P* < 2 × 10^−16^). To illustrate the potential value of computational pathology in providing prognostic information about hepatic decompensation, maximally selected rank statistics were used to determine the optimal PSR percentage cutpoint that divided samples into high- and low-risk groups; the Kaplan–Meier estimator curves are shown in Extended Data Fig. 1i.

Finally, the development of HCC is a low-frequency outcome in MASLD. The complete SteatoSITE cohort includes 80 patients with a coding of HCC at any point in their annotated timeline. The increased risk of HCC in patients with non-cirrhotic MASLD is also well-recognized^23,24^. The SteatoSITE cohort includes 227 resection specimens, with HCC being present in the annotated patient timeline in 44 of these. Notably, 36 of these cases did not have histopathological evidence of cirrhosis in the background liver (Extended Data Fig. 1j). Focusing only on the liver biopsy cases in which no event coding for HCC was present before the biopsy, ten patients received a subsequent coding of HCC in the follow-up period included in the data commons, and assigned NASH-CRN F4 fibrosis stage at biopsy was predictive of the subsequent development of HCC (Extended Data Fig. 1k).

### Transcriptomic profiling

The SteatoSITE cohort includes high-quality hepatic RNA-seq data in 668 out of the 940 total cases (comprising 538 biopsies, 39 explants and 130 resections), after applying prespecified quality-control criteria appropriate for archival formalin-fixed paraffin-embedded (FFPE) samples, including the percentage of RNA fragments > 200 nucleotides (DV_200_ ) > 30% (ref. ^25^). Overall, larger liver-resection tissues yielded poorer RNA quality compared with much smaller needle biopsy specimens, likely related to sample fixation.

**Extended Data Fig. 2:**
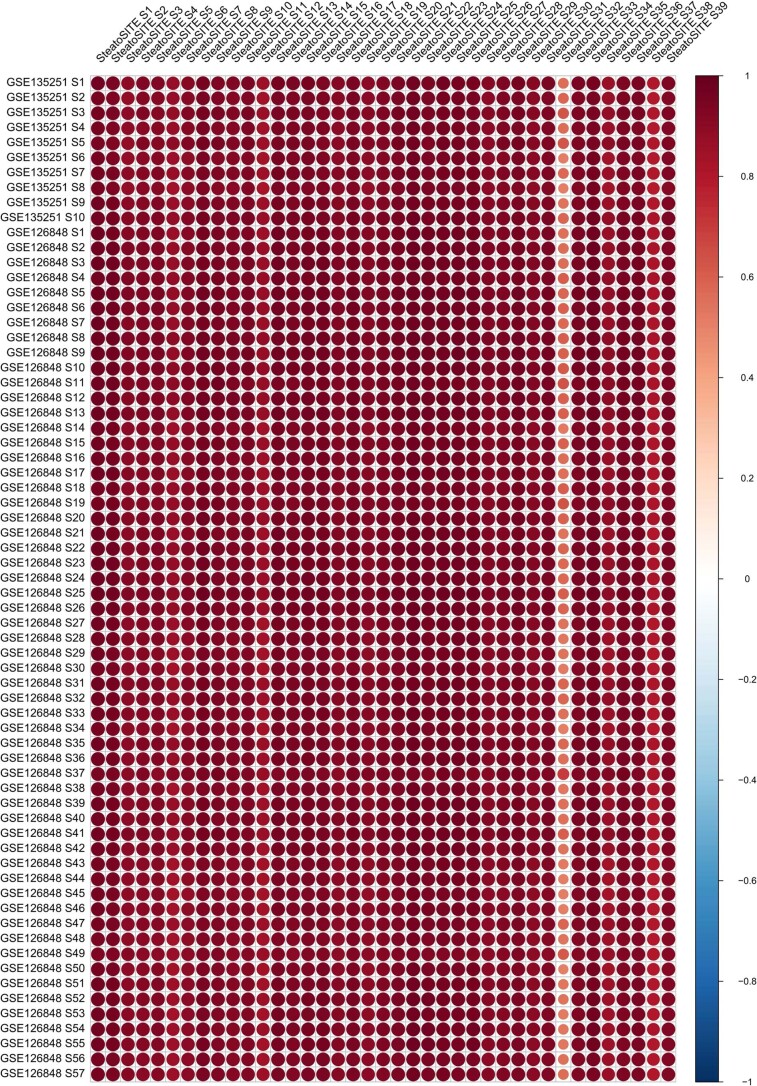
Validation of normal liver transcriptome. Correlation (corrplot) of transcriptomic profiles of normal SteatoSITE samples (*n* = 39) with published normal liver reference data (*n* = 57). The colour scale corresponds to the strength and direction of correlations.

Normal liver controls (*n* = 39) were also retrieved from the biorepositories for comparative RNA-seq analysis. To confirm the suitability of these control samples, we showed that their transcriptional profile strongly correlated with normal liver samples from independent hepatic RNA-seq datasets^26,27^ (average *r*_s_ > 0.9, adjusted *P* < 2 × 10^−16^; Extended Data Fig. 2). After applying quality-control checks, 34 control samples were used for further analyses.

We performed RNA-seq variant calling to detect the most relevant and replicated single-nucleotide polymorphisms (SNPs) identified as risk modifiers of MASLD progression (rs738409, rs72613567, rs58542926 and rs641738 in the genes patatin-like phospholipase domain containing 3 (*PNPLA3*), hydroxysteroid 17-beta dehydrogenase 13 (*HSD17B13*), transmembrane 6 superfamily member 2 (*TM6SF2*) and membrane-bound O-acyltransferase domain containing 7 (*MBOAT7*), respectively). The SNP frequency across histological fibrosis stages is shown in Supplementary Table 11. Sequencing coverage of these genes, and therefore the ability to call variants, was variable. However, the rs738409 C>G p.I148M variant in *PNPLA3* (the strongest genetic risk factor for MASLD and its severity) was successfully called in 612 of 668 MASLD samples with RNA-seq available. The prevalence of GG, GC and CC genotypes among cases where a call was possible was 16.8%, 27.8% and 54.7%, respectively. After controlling for fibrosis stage, genotype status for predefined variants had no substantial effect on outcomes (data not shown), consistent with findings from a previous long-term follow-up study of 901 individuals with MASLD in which overall mortality was not affected by any genetic variant^28^.

**Fig. 2:**
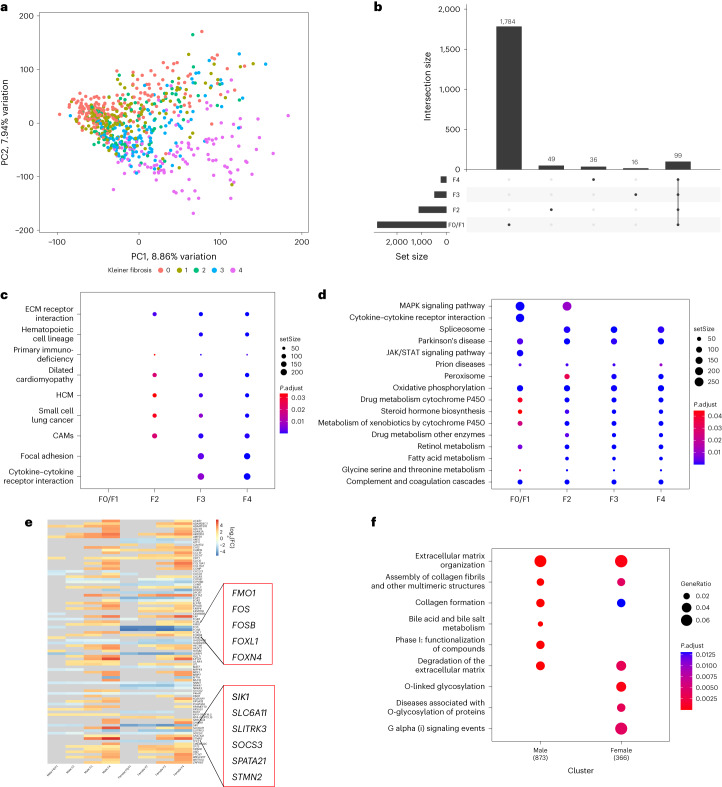
Hepatic transcriptomic analysis and summary bioinformatics. **a**, PCA plot for 616 MASLD samples and 34 normal liver control samples after including batch effects as covariates in the linear model. The first PC is displayed on the *x* axis and the second PC on the *y* axis, with the corresponding percentage of total variance explained by each PC. Dots represent individual samples colored according to NASH-CRN fibrosis stage. **b**, UpSet plot of DEGs showing the unique DEGs belonging to individual sets (fibrosis stages F0/1, F2, F3, F4) and the intersection of DEGs across all fibrosis stages. Set sizes are presented as bars, and their composition is described by the bottom panel. **c**,**d**, Dot plots showing the downregulated (**c**) and upregulated (**d**) Kyoto Encyclopedia of Genes and Genomes pathways (Benjamini and Hochberg false discovery rate *q* < 0.05 and fold-change of ≥1) obtained from one-sided gene-set-enrichment analysis for different fibrosis stages (F0/1, F2, F3, F4). The size of the dot is on the basis of gene count enriched in the pathway, and the color of the dot shows the pathway enrichment significance. **e**, FC of the top 20 differentially expressed genes in each fibrotic stage for both men and women. Exemplar dysregulated genes between men and women are highlighted for illustration. The gray color indicates that the genes were not statistically significantly dysregulated for that specific stage. **f**, Dot plot highlighting the differences between the Reactome pathways (*q* < 0.05 and FC ≥1) obtained by one-sided GSEA in men and women in stage F4 compared with controls. setSIZE indicates the total number of genes present in each specific gene set. P.adjust indicates the Benjamini and Hochberg adjusted *P* values. GeneRatio is *k*/*n*, where *k* is the overlap between the genes of interest and the gene set and *n* is the number of all unique genes of interest. CAM, cell adhesion molecule; ECM, extracellular matrix; HCM, hypertrophic cardiomyopathy; JAK, Janus kinase; MAPK, mitogen-activated protein kinase; STAT, signal transducer and activator of transcription.

For differential gene expression analysis, samples were grouped according to NAS and different fibrosis stages (F0/F1, F2, F3 and F4) and compared with normal liver control samples. The data structure was examined by principal component analysis (PCA). As shown in Fig. 2a, the first two principal components (PCs) explained 8.86% and 7.94% of the observed variation in gene expression. There was modest segregation according to the fibrosis stage.

When samples were classified according to fibrosis stage, we found 218, 478, 1,114 and 2,800 differentially expressed protein-coding genes when comparing F0/F1, F2, F3 and F4 livers with normal liver controls, respectively, and 99 genes differentially expressed in common across stages versus controls (Fig. 2b).

Kyoto Encyclopedia of Genes and Genomes pathways linked to extracellular matrix were enriched from stage F2 onwards, as expected. Notably, other upregulated pathways were those linked to cardiomyopathies (*q* < 0.05; Fig. 2c). In contrast, downregulated genes were enriched for pathways associated with signaling, predominantly in early disease (F0/1), and fatty acid metabolism and peroxisome pathways were enriched from F2 to F4 (*q* < 0.05; Fig. 2d).

**Extended Data Fig. 3:**
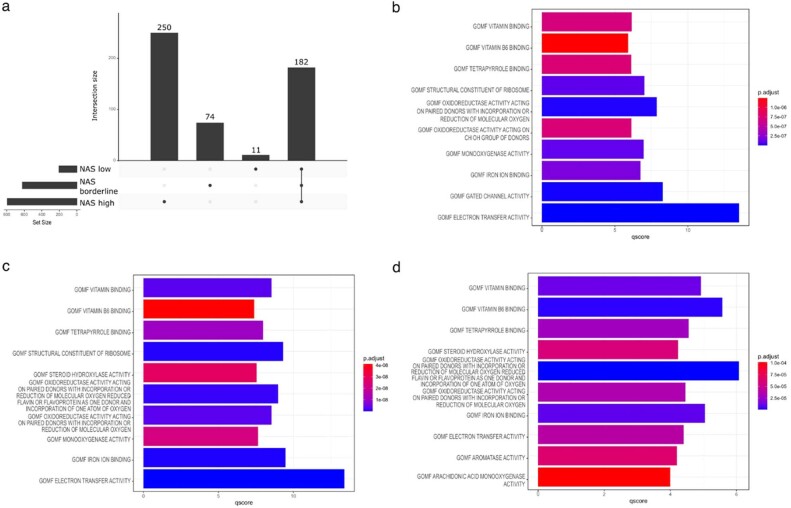
SteatoSITE RNA-seq according to NAS scores. **a**, UpSet plot of differentially assessed genes (DEGs), showing DEGs belonging to individual sets (according to NAFLD activity score (NAS) ≤ 2, NAS 3/4 and NAS ≥ 5) and the intersection of DEGs across all NAS sets. Set sizes are presented as bars and their composition is described by the bottom panel. **b**, Top 10 molecular functions (gene ontology (GO) terms) identified with one-sided gene set enrichment analysis based on the differentially expressed genes of NAS ≤ 2; **c**, NAS 3/4; **d**, NAS ≥ 5 (Benjamin and Hochberg false discovery rate q < 0.05 and fold-change of ≥1).

When the samples were grouped according to NAS, we identified 203 differentially expressed genes when comparing NAS ≤ 2 (‘low NAS’) with controls, 621 in the NAS 3–4 group (‘borderline NAS’) and 793 in the NAS > 4 group (‘high NAS’); 182 genes were shared across all NAS groups (Extended Data Fig. 3a). Gene set enrichment analysis (GSEA) revealed, among others, significantly enriched gene ontology terms that participate in ‘translation’ and the ‘immune system’ (*q* < 0.05; Extended Data Fig. 3b–d).

**Extended Data Fig. 4:**
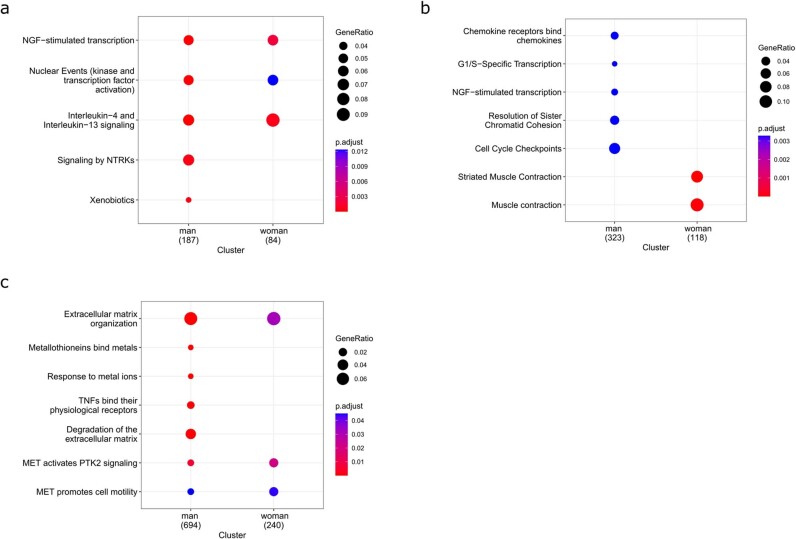
SteatoSITE RNA-seq gender-focused gene enrichment analysis. Reactome pathways obtained from one-sided gene set enrichment analysis in both men and women for **a**, F0/1 vs control; **b**, F2 vs controls; **c**, F3 vs controls (Benjamin and Hochberg false discovery rate q < 0.05 and fold-change ≥ 1).

As previous studies have highlighted the sexually dimorphic nature of MASLD^29^, we also determined gender-specific differences in gene expression and biological pathway enrichment across the MASLD histological spectrum. After stratifying patients by gender and fibrosis stage, we performed differential gene expression analysis to discern shared and gender-specific molecular profiles. In men, we identified 156, 253, 1,167 and 2,767 differentially expressed genes in F0/F1, F2, F3 and F4, respectively, when compared with controls. In women, we identified 383, 768, 1,285 and 2,997 genes in each fibrosis stage, respectively. This indicates that there are more dysregulated genes in women than men. The top 20 differentially expressed genes in each fibrosis stage for both genders are presented in Fig. 2e. Additionally, we performed enrichment analysis between men and women at each stage of fibrosis and observed gender-specific differences in biological pathways. To illustrate, in Fig. 2f, we show that for stage F4, most of the identified pathways are common to both genders, apart from ‘bile acid and bile salt metabolism’ and ‘phase I-functionalization of compounds’, which are only present in men. The enriched pathways obtained in other fibrosis stages are shown in Extended Data Fig. 4.

Hence, SteatoSITE enables a comparison of whole-liver gene expression profiles according to histological disease stage. To maximize the accessibility and utility of this resource, we developed an open-access gene browser (https://shiny.igc.ed.ac.uk/SteatoSITE_gene_explorer/), allowing high-level assessment and visualizations of user-selected gene expression according to fibrosis stage.

### Cell-type characterization by single-cell deconvolution

Using single-cell RNA-seq (scRNA-seq), we have previously identified distinct populations of scar-associated monocyte-derived macrophages, mesenchymal cells and endothelial cells that populate the fibrotic niche in patients with advanced cirrhosis and interact to regulate liver fibrogenesis^30^. However, the presence of specific pathogenic cellular subpopulations across the full MASLD disease spectrum and how these cells relate to clinical outcomes remain uncertain. To address this, we performed deconvolution of the SteatoSITE bulk RNA-seq data using a publicly available fully annotated scRNA-seq reference dataset compiled from healthy and cirrhotic patients^30^ to estimate the proportions of specific hepatic cell types in each SteatoSITE sample and correlate them with histopathological features and patient outcomes.

**Fig. 3:**
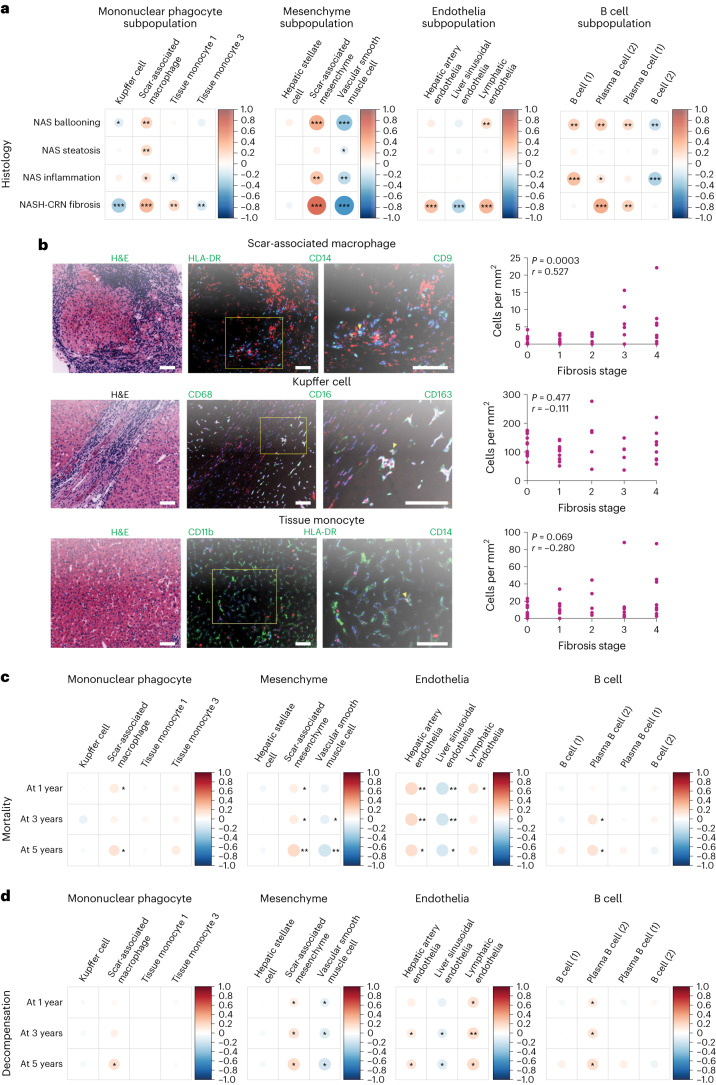
Hepatic bulk RNA-seq data with clinical annotation. **a**, Corrplots of macrophage, mesenchyme, endothelia and B cell subpopulation proportion correlations with NAS components and NASH-CRN fibrosis stage. *P < 0.05, **P < 0.01, ***P < 1 × 10^−6^. Color and size of circles indicate the Pearson correlation coefficients. **b**, Representative H&E-stained sections and multiplex immunofluorescent staining to identify subpopulations of SAMacs, Kupffer cells and tissue monocytes. Selected markers shown. Arrowheads indicate stated cell type. Correlation of number of stated cell type per mm^2^ tissue with fibrosis stage (*n* = 43; Spearman’s rho and *P* value indicated). Scale bars, 100 µm. **c**,**d**, Corrplots of macrophage, mesenchyme, endothelia and B cell subpopulation proportion correlations with 1-, 3- and 5-year all-cause mortality (**c**) and decompensation risk (**d**). *P < 0.05, **P < 0.01. Color and size of circles indicate the Pearson correlation coefficients.

**Extended Data Fig. 5:**
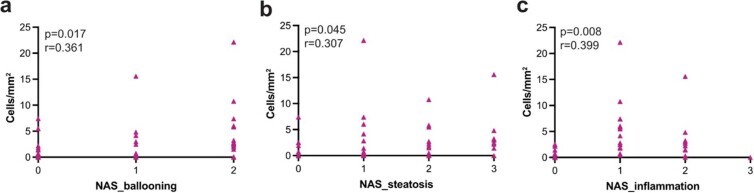
Correlation of scar-associated macrophage numbers with NAFLD activity score components. Scar-associated macrophage numbers determined by multiplex immunofluorescence correlated positively with assigned components of the NAFLD activity score (n = 43). Spearman’s rho and *P*-value indicated for ballooning (**a**), steatosis (**b**), and inflammation (**c**).

This analysis demonstrated that the proportion of hepatic scar-associated macrophages (SAMacs), a key regulator of liver fibrosis^30^, correlated positively with liver fibrosis stage and steatohepatitis activity across the full MASLD spectrum (Fig. 3a). In contrast, tissue-resident macrophage (Kupffer cell) proportions declined substantially with more advanced fibrosis (Fig. 3a). We validated these transcriptomic findings at the protein level using a bespoke MultiOmyx liver multiplex immunofluorescence (mIF) assay in an independent MASLD histological dataset (*n* = 43), confirming a statistically significant positive correlation of hepatic HLA-DR^+^CD9^+^CD14^+^ SAMac numbers with liver fibrosis stage (Fig. 3b). Positive correlations between mIF SAMac numbers and steatosis, lobular inflammation and ballooning scores were less pronounced than fibrosis stage (Extended Data Fig. 5a–c), mirroring our findings from the deconvolution analysis (Fig. 3a).

Deconvolution analysis also revealed statistically significant positive correlations between fibrosis and the proportion of scar-associated mesenchymal cells, hepatic arterial endothelial cells and lymphatic endothelial cells (Fig. 3a), in keeping with the key roles for mesenchymal cell activation and progressive arterialization of the hepatic microcirculation with loss of normal specialized liver sinusoidal endothelial cell phenotype (‘capillarization’) in the development of liver fibrosis across the full MASLD spectrum. Interestingly, expansion of plasma cells was also associated with more advanced hepatic fibrosis (Fig. 3a).

Finally, the proportions of cell types, derived by single-cell deconvolution analysis of SteatoSITE bulk RNA-seq data, were examined for their value in predicting adverse clinical outcomes. Strikingly, increased hepatic proportions of SAMacs, scar-associated mesenchymal cells, hepatic arterial endothelial cells, lymphatic endothelial cells and plasma cells were predictive of higher future all-cause mortality (Fig. 3c) and hepatic decompensation events (Fig. 3d). In contrast, higher proportions of more homeostatic liver resident cell types such as vascular smooth muscle cells and liver sinusoidal endothelial cells were protective against future mortality or hepatic decompensation (Fig. 3c,d). Hence, in addition to histology and bulk transcriptomics, changes in the cellular composition of the liver may offer key prognostic information in patients with MASLD.

### Transcriptional risk prediction of hepatic decompensation

The annotated patient timelines in SteatoSITE allow predictive tools to be developed. To demonstrate, we used the SteatoSITE RNA-seq data and associated clinical outcomes to develop a novel transcriptome-based risk-prediction model for hepatic decompensation. Such transcriptional risk scores (TRSs) on the basis of transcript abundance are physiologically closer to the phenotype of interest, require smaller training samples and offer greater portability across diverse ancestry groups than polygenic risk scores using genomic variants (SNPs)^31,32^.

**Extended Data Fig. 6:**
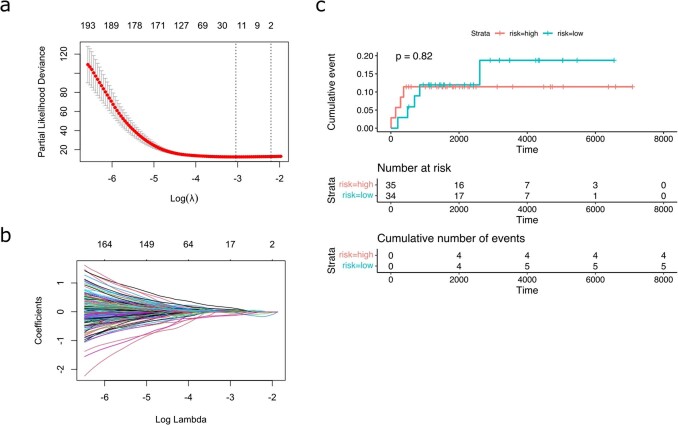
Regularised Cox regression model. **a**, Plot of the optimal value of logarithmic lambda (*n* = 100) and the cross-validation error. Data presented as mean value +/− SD. **b**, Coefficient values across the logarithmic lambda. **c**, Kaplan-Meier plot demonstrating no separation of the event curves of patients with biopsies showing F2 fibrosis using the transcriptional risk score; log rank test *P*-value = 0.82.

**Extended Data Fig. 7:**
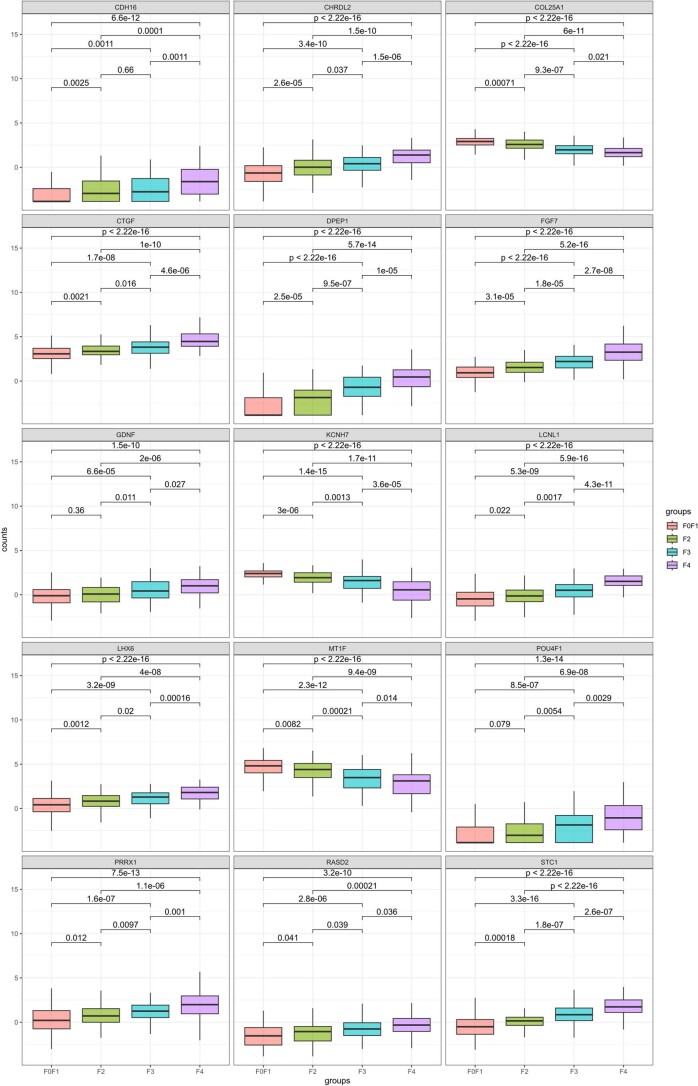
Gene expression of the prognostic signature across the fibrotic stages. Plots of the normalised expression of the named 15 gene constituents of the transcriptional risk score by NASH-CRN fibrosis stage (*n* = 368), as visualised by the Shiny transcriptome browser app. All boxplots show median (centre line), first and third quartiles (lower and upper box limits), 1.5× interquartile range (whiskers). *P*-values of one-way ANOVA with post-hoc Tukey test.

As the initial feature set, we used the 1,127 protein-encoding genes that were differentially expressed in advanced (F3/F4) compared with early (F0/F1) stage disease (*P* < 0.05, log fold change (FC) ≥1). Univariate Cox regression identified 955 differentially assessed genes (DEGs) as significantly related to decompensation events (*P* < 0.01). To develop a sparse feature set, ten runs of a tenfold lasso-regularized Cox regression were performed, and the coefficients for selected genes were derived (Extended Data Fig. 6a,b). The final TRS predicting hepatic decompensation was composed of the expression of 15 genes: metallothionein 1F (*MT1F*), potassium voltage-gated channel subfamily H member 7 (*KCNH7*), collagen type XXV alpha 1 chain (*COL25A1*), RASD family member 2 (*RASD2*), connective tissue growth factor (*CTGF*), stanniocalcin 1 (*STC1*), glial-cell-derived neurotrophic factor (*GDNF*), paired related homeobox 1 (*PRRX1*), fibroblast growth factor 7 (*FGF7*), lipocalin-like 1 (*LCNL1*), dipeptidase 1 (*DPEP1*), chordin-like 2 (*CHRDL2*), LIM homeobox 6 (*LHX6*), POU class 4 homeobox 1 (*POU4F1*) and cadherin 16 (*CDH16*). (Expression across fibrosis stages as plotted by the SteatoSITE gene browser is shown in Extended Data Fig. 7.)

**Fig. 4:**
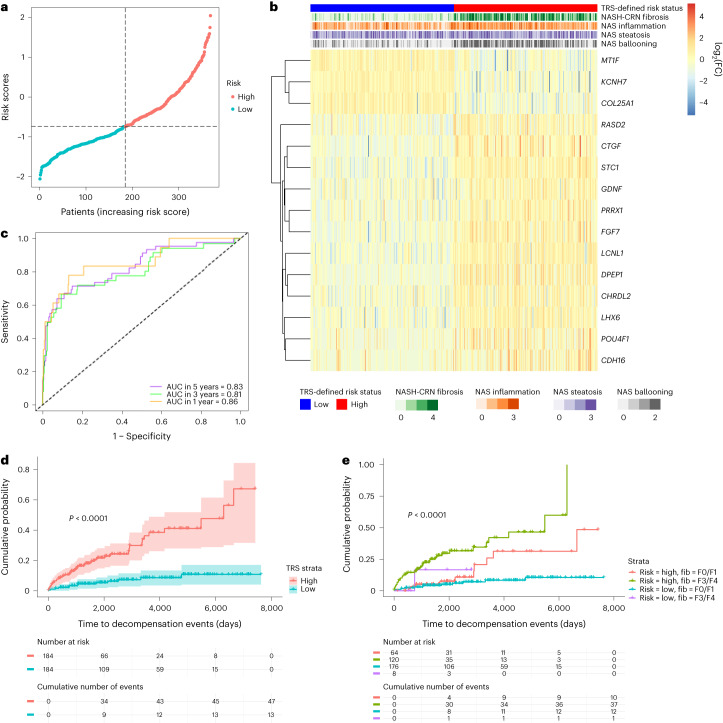
Prediction of risk of future decompensation events in advanced fibrosis using hepatic bulk RNA-seq data. **a**, Correlation between the risk scores and the time of the decompensation events. **b**, Heatmap of the gene expression profile of the prognostic signature. **c**, Time-dependent ROC curves and AUC analyses by the expression of the 15 genes. **d**, Kaplan–Meier curves (with 95% confidence intervals) showing high- and low-risk groups for all biopsy cases; log-rank test *P* value < 0.0001. **e**, Kaplan–Meier curves demonstrating separation of patients into high- and low-risk groups in both mild (F0/1) and severe (F3/4) fibrosis stages; log-rank test *P* value.

The risk scores were calculated using the formula indicated in the Methods. According to the median risk score, patients were split into high- and low-risk groups (Fig. 4a). Interestingly, samples with a higher risk score had not only a higher fibrosis stage but also a higher hepatocyte ballooning score (Fig. 4b). Time-dependent receiver operating characteristic (ROC) curves were used to assess the predictive ability of the model at specific times after biopsy; the areas under the ROC curves (AUCs) were 86.24% (standard error (SE) 5.11), 80.97% (SE 4.62) and 83.26% (SE 3.86) for 1-, 3- and 5-year risk of decompensation events, respectively (Fig. 4c).

All the biopsy samples could be stratified using the TRS into high- and low-risk groups with substantially different decompensation trajectories. Over postbiopsy follow-up of up to 20 years, those with a high-risk TRS had a cumulative decompensation event probability of more than 0.65 compared with a cumulative probability of 0.11 in those with a low-risk TRS (Fig. 4d). Next, to derive further prognostic information beyond routine fibrosis staging, samples with mild (F0/F1) or advanced (F3/F4) scarring were stratified using the TRS into groups at high or low risk of future decompensation (Fig. 4e). Although there were insufficient F3/F4 patients with a low TRS to enable further categorization of these patients, application of the TRS augmented risk stratification in MASH patients with early-stage fibrosis. Stratification of biopsies with F2 fibrosis is shown in Extended Data Fig. 6c.

### Master regulator analysis of disease progression

**Fig. 5:**
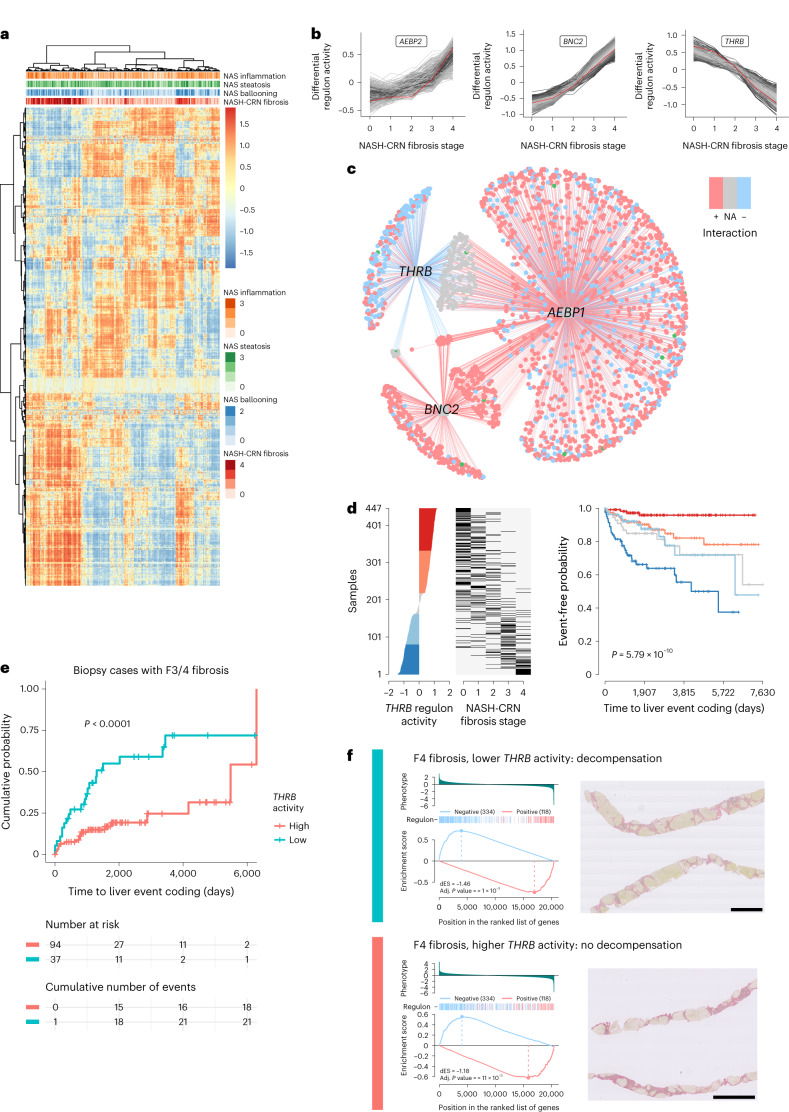
The relationship of *THRB* with disease progression. **a**, Heatmap of estimated transcriptional network (regulon) activity across the SteatoSITE dataset. **b**, Patterns of activity and cluster membership of the three regulons (*THRB*, *AEBP2*, *BNC2*). **c**, Relationships of *THRB*, *AEBP1*, *BNC2* regulatory networks with significant numbers of component genes from the TRS as gene target members in disease progression. Red edges, positive regulation of gene target; blue edges, negative regulation of gene target; blue nodes, net negative gene regulation; red nodes, net positive gene regulation; gray nodes, net neutral gene regulation; green nodes, TRS gene member. **d**, Ranked *THRB* regulon differential activity plot confirms the negative relationship between *THRB* regulon activity and disease stage (left and center), and the Kaplan–Meier plot indicates that lower *THRB* regulon activity is predictive of hepatic decompensation (right); log-rank test *P* value. **e**, In histologically identical high-risk fibrosis stages (F3 and F4), low *THRB* activity identifies patients at high risk of hepatic decompensation event; log-rank test *P* value < 0.0001. **f**, Example individual two-tailed gene set enrichment plots of target genes in the *THRB* regulon (left, two-tailed gene set enrichment testing with Benjamini and Hochberg (false discovery rate) adjusted *P* values < 0.001) from two patients with identical F4 stage (cirrhosis) on PSR-stained sections (right), where the patient with low *THRB* differential regulon activity (top, blue) progressed to a hepatic decompensation event 224 days after biopsy in contrast to a patient with high *THRB* regulon activity who did not experience a decompensation event during the 4,500 days until censoring (bottom, red). Scale bars, 2 mm. dES, differential enrichment score.

We sought to further exploit the rich RNA-seq dataset using the high-risk genes identified as prognostically important components of the TRS to derive further biological understanding of the related transcriptional regulatory network (TRN) in MASLD. The TRN, consisting of transcription factors and regulated target genes, was inferred from the whole-gene-expression dataset. Regulons, a set of genes regulated by a specific transcription factor, were constructed for all transcription factors cataloged by ref. ^33^. A regulon activity score for each sample was estimated using a two-tailed GSEA approach. Regulons cluster on the basis of activity into two broad groups: those with high activity in advanced fibrosis and low activity in early stages and those showing the opposite pattern (Fig. 5a).

To identify which transcriptional networks lie upstream of the expression of high-risk genes representing the TRS, master regulator analysis^34^ was undertaken to identify the statistical significance of the overlap between the regulon (gene targets) of each transcription factor and the 15 genes of the TRS, corrected for multiple hypothesis testing. Three regulons (gene networks regulated by AE binding protein 1 (*AEBP1*), thyroid hormone receptor beta (*THRB*) and zinc finger protein basonuclin zinc finger protein 2 (*BNC2*)) contained substantially greater numbers of the TRS genes than expected by chance, indicating that these three networks may be critical to MASLD disease progression leading to decompensation events.

To examine the patterns of activity of these regulons during progression of disease stage, the mean activity scores for each regulon from each NASH-CRN fibrosis stage were used as pseudo-timeseries data for unsupervised soft clustering. AE binding protein 2 (*AEBP2*) regulon activity is in a cluster of regulons whose activity is low in F0–2 but increased in F3 and F4, whereas *BNC2* regulon activity increases more uniformly from F0 to F4. In contrast, *THRB* regulon activity is high in F0 and F1 but decreases as scarring progresses from F2 to F4 (Fig. 5b).

The relationship between the inferred *AEBP1*, *THRB* and *BNC2* regulons is shown in Fig. 5c, annotated with the direction of regulation of each gene target. Most gene targets for *AEBP1* and *BNC2* are positively regulated, whereas *THRB* exerts largely negative regulation of gene targets shared with both *AEBP1* and *BNC2*.

Given the potential suppressive role for *THRB* over disease-promoting core gene targets, and because *THRB* agonists are in current clinical trials for MASLD, we examined *THRB* regulon activity in more detail. In biopsy cases with no hepatic decompensation before biopsy, differential regulon activity was estimated and the predictive value of *THRB* regulon activity for decompensation events examined (Fig. 5d). A ranked *THRB* regulon differential activity plot confirms the negative relationship between *THRB* regulon activity and disease stage in the biopsy-restricted subset, and the Kaplan–Meier plot indicates that lower *THRB* regulon activity is predictive of hepatic decompensation.

**Extended Data Fig. 8:**
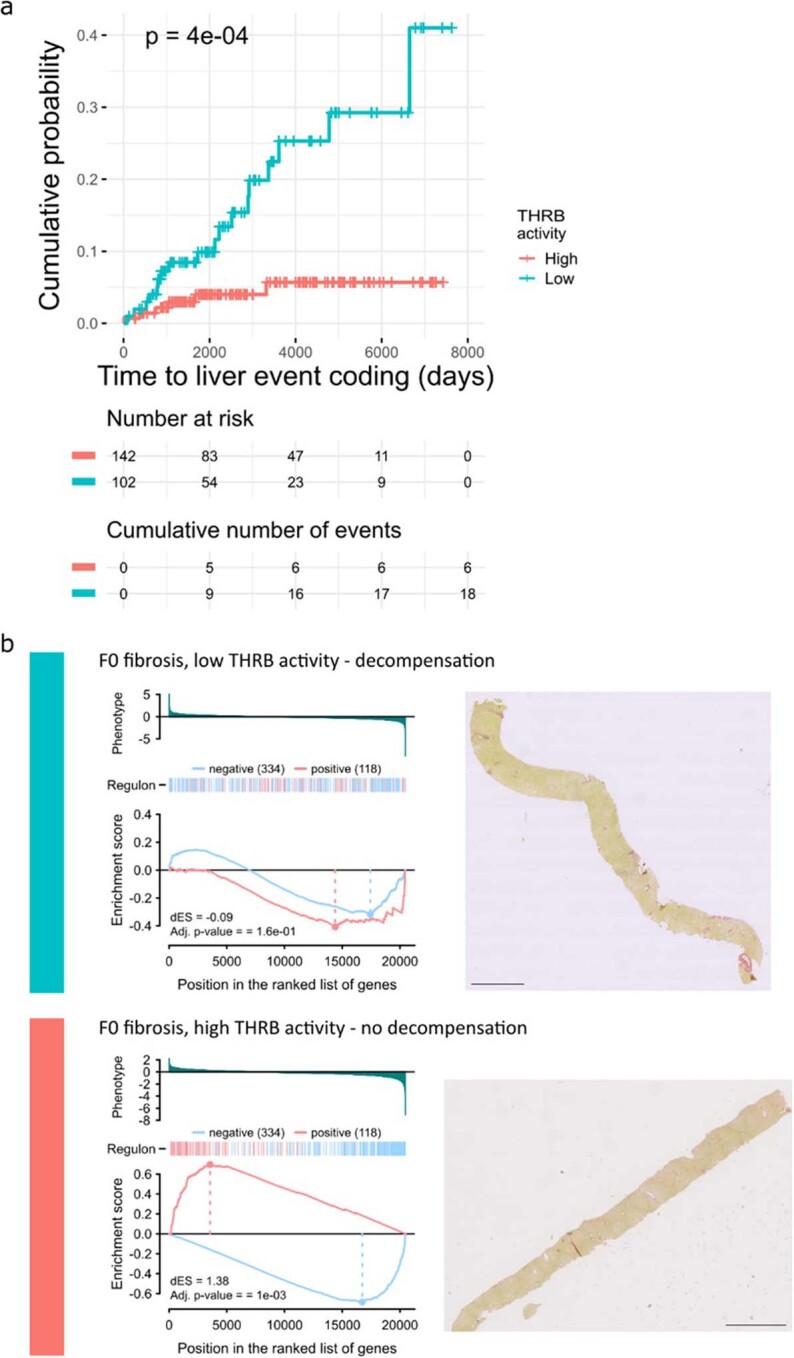
Additional predictive and personalisation value of estimated THRB regulon activity in F0 and F1 stage disease. **a**, In histologically identical low-risk fibrosis stages (F0 and F1 biopsy cases), low THRB activity identifies patients at high risk of progression to a hepatic decompensation event; log rank test *P*-value. **b**, Example individual 2-tailed gene set enrichment plots of target genes in the THRB regulon (left, 2-tailed gene set enrichment testing with Benjamini and Hochberg (false discovery rate) adjusted *P*-values) from two patients with identical F0 stage (no scarring) on PSR stained sections (right), where the patient with low THRB differential regulon activity (top, blue) progressed to a hepatic decompensation event 3000 days after biopsy in contrast to a patient with high THRB regulon activity who did not experience a decompensation event during the 4400 days until censoring (bottom, red). Scale bars, 2 mm.

However, the predictive value of *THRB* regulon activity may only reflect association with disease stage. To determine whether *THRB* regulon activity was predictive of hepatic decompensation in samples of matched fibrosis stages, maximally selected rank statistics were used to determine the optimal *THRB* regulon activity cutpoint that divided samples into high- and low-risk groups. Using this cutpoint on histologically low-risk samples with F0 and F1 fibrosis, *THRB* regulon activity identified a subset of MASLD patients with high risk of hepatic decompensation (Extended Data Fig. 8). Deriving a new cutpoint in only the histologically advanced stages F3 and F4 allowed stratification into two distinct groups with either rapid or slower progression to a decompensation event (Fig. 5e).

The potential for a personalized approach, for clinical trials or routine practice, using estimated *THRB* regulon activity is illustrated by case studies from the SteatoSITE dataset. Two cases scored F4 (cirrhosis) on PSR-stained sections with high and lower estimated *THRB* activity are shown in Fig. 5f; the patient with estimated *THRB* regulon activity below the derived threshold (top) had a coded decompensation event 224 days after biopsy, but the patient with *THRB* activity above the cutpoint (bottom) did not experience a coded decompensation event in more than 4,500 days of follow-up. At the other end of the disease stage spectrum, two cases that scored F0 on the PSR-stained sections are shown in Extended Data Fig. 8; the case with high estimated *THRB* regulon activity derived from RNA-seq (bottom) was not associated with hepatic decompensation, whereas the case with low estimated *THRB* regulon activity (top) was.

## Discussion

We have established what is to the best of our knowledge a unique resource comprising extensive histopathological assessments, transcriptome analysis and rich clinical data across the full MASLD severity spectrum. SteatoSITE is a dataset of broad utility that will enable discovery science, digital pathology research, artificial intelligence approaches and translational studies in MASLD (a subset of the umbrella grouping of steatotic liver disease (SLD)) in the future. Our approach is complementary to continuing global initiatives in MASLD such as biomarker-focused research being conducted by the Liver Investigation: Testing Marker Utility in Steatohepatitis and Non-Invasive BioMarkers of MetaBolic Liver DiseasE^35^ consortia, as well as ‘omics repositories such as http://liverproteome.org/ and https://www.livercellatlas.org/index.php that contain MASLD and human subsections, respectively. However, crucially, gene signatures and histopathological metrics in SteatoSITE are linked to clinical outcomes to enable rational diagnostic and treatment approaches for patients with different stages and activity of disease to be determined. Indeed, an authoritative expert review stated that “for more refined treatment of NASH, orthogonal approaches that integrate genetic, clinical and pathological datasets may yield treatments for specific subphenotypes of the disease”^36^. We believe that SteatoSITE provides the relevant multiscale and multimodal data required to meet this challenge.

Consistent with previously published observational data^37^, we began by showing the strong positive association between histological fibrosis stage and future clinical outcomes including all-cause mortality, HCC and a composite outcome of hepatic decompensation events. These findings validated the broad content and data integration of the SteatoSITE cohort. We went on to perform a comprehensive bioinformatic analysis of the hepatic bulk RNA-seq data, identifying key genes and enriched gene functions/pathways that characterize discrete histological subtypes and generating disease stage-specific transcriptional profiles to inform drug-target discovery and the development of clinically relevant biomarkers in MASLD. In addition, we provide an open-access web-based gene browser so the scientific community can interactively explore and visualize the SteatoSITE RNA-seq data, thus increasing the accessibility and effect of our work.

As studies have highlighted the sexually dimorphic nature of MASLD^38^, we also performed gender-specific analyses to explore differences in gene expression and molecular pathways. Across the MASLD disease spectrum, we identified transcriptional differences and gender-biased enrichment of molecular pathways, such as bile acid and bile salt metabolism and O-linked glycosylation in stage F4 livers. Further work is needed to determine the effect of such differences on disease progression in MASLD, as well as their relevance to biomarkers, therapeutic targets and treatment responses in men and women.

The advent of single-cell transcriptomic technologies is transforming our understanding of the pathobiology of liver diseases including MASLD, identifying key pathogenic cell types and candidate therapeutic targets^39–41^. However, the study of these cell populations across the full disease spectrum remains limited, with minimal data defining which specific cell states are associated with adverse clinical outcomes. Here, we used a single-cell deconvolution approach to interrogate changes in cellular composition in the SteatoSITE cohort, confirming expansion of SAMacs, scar-associated mesenchymal cells, plasma cells, hepatic arterial endothelial cells and lymphatic endothelial cells during MASLD progression. Crucially, expansion of these populations also correlates with poor long-term patient prognosis, highlighting their potential role as therapeutic targets in MASLD and informing novel immunohistochemical approaches to better characterize liver biopsy tissue and identify high-risk patient subphenotypes. Indeed, multiplex immunohistochemistry is already being used to improve stratification in patients with a range of cancers^42–44^, so future refinement of our liver macrophage-focused high-plex MultiOmyx assay to include specific markers for pathogenic mesenchymal, endothelial and plasma cells will enable a comprehensive assessment of the liver fibrotic niche from a single slide and may facilitate improved identification of high-risk MASLD patients compared to existing histological approaches.

As the individual patient-level datapoints in SteatoSITE are temporally defined, researchers can perform time-to-event predictions using survival-analysis methods. Here, we illustrated this by showing that hepatic gene expression data in our cohort had a high predictive value for risk of future hepatic decompensation events, over and above standard fibrosis staging. The transition from compensated cirrhosis to decompensated cirrhosis occurs at a rate of about 5% to 7% per year^45^. Decompensation represents a key prognostic inflection point in the natural history of chronic liver disease, as the median survival drops from more than 12 years for compensated cirrhosis to about 2 years for decompensated cirrhosis^45^. However, in patients with advanced fibrosis or cirrhosis (F3/F4), individual risk of decompensation is variable and hard to predict, and preventative therapies are lacking. On the basis of automated variable selection methods to reduce overfitting, we identified a 15-gene panel and derived a risk score that could classify stage-defined MASLD patients into high- or low-risk groups for decompensation^46,47^. To the best of our knowledge, there are no TRSs reported in MASLD; but notably, a TRS approach outperformed static genotypic risk assessment in distinguishing Crohn’s disease from healthy samples and predicting complications^48^. Genes represented in our TRS include five members predicted to be secreted proteins, according to The Human Protein Atlas (https://www.proteinatlas.org), which could be explored further in suitably well-annotated clinical samples. Although not previously reported in MASLD, *CHRDL2* is upregulated in a range of tumor tissues including HCC^49^, while *STC1*, *CTGF*^50^, *GDNF* and *FGF7* are all linked to hepatic fibrogenesis^51,52^. Additionally, *STC1* encodes a secreted glycoprotein reported as a potential serum biomarker for hepatitis B virus-associated liver fibrosis^51^, and secreted *FGF7* protein^53^ was identified as a prognostic marker in cholangiocarcinoma. The predictive model requires external validation, ideally in prospective longitudinal studies. However, to our knowledge, there are at present no available MASLD datasets with integrated pathology, transcriptomics and clinical outcome data to enable this.

Finally, to search for new and highly effective upstream therapeutic targets, we used high-risk genes from the TRS to uncover core gene networks and master regulators likely to exert influence over disease progression and patient outcomes. Intriguingly, one regulon—*THRB*—was suppressive of the other two identified (*AEBP1* and *BCN2*, which are linked to hepatic fibrogenesis and MASLD progression^54,55^). Moreover, *THRB* regulon activity not only decreased with advancing fibrosis stage but also predicted future hepatic decompensation (beyond standard fibrosis scoring). Liver-targeted thyromimetics selectively activating *THRB*, such as resmetirom (MGL-3196) and VK2809, increase hepatic fat metabolism and reduce lipotoxicity and have recently emerged as leading pharmacological candidates for the treatment of MASLD^56^. Indeed, resmetirom^57^ has advanced to phase 3 trials^58^, and a MASLD cirrhosis-outcomes trial is underway. Our data reinforce the importance of *THRB* as a therapeutic target with the potential to affect hard clinical endpoints and illustrate how adding transcriptomic information to histology might further stratify prognosis and enable tailoring of treatment.

The creation of SteatoSITE was facilitated by the Scottish demographics and healthcare infrastructure. Multimodal data were drawn from three of the four regional Safe Havens (trusted research environments), covering 12 of the 14 territorial Health Boards, and thus sampling most of the Scottish population. Scotland has a well-established ecosystem for precision medicine consisting of a stable population base of ~5.5 million people, a significant incidence of major chronic disease (death rates from chronic liver disease in Scotland are 70% higher than the UK average^59^), a single healthcare provider (National Health Service (NHS) Scotland) and an EHR system operating on a national scale with a unique common field identifier (Community Health Index (CHI) number) allowing characterization and longitudinal follow-up of well-defined patient cohorts; these key elements differentiate Scotland from many other countries.

Given the scale and complexity of SteatoSITE and its use of routine retrospective multicentric clinical data, several technical and organizational challenges and limitations are acknowledged. Leveraging EHR data for MASLD research is recognized as potentially powerful, but consensus guidelines have only emerged over the past three years^21,60^. Using three different NHS Safe Havens necessitated an exacting and systematic data-cleaning process, including data duplication, human error (for example, incorrect data entry, typographical errors, sample mislabeling), issues with data standardization, errors due to different delimiters/encoding in input files, data formatting and missing data/completeness. Notably, some source data (for example, FibroScan results) are inconsistently recorded across Health Boards and were therefore hard to obtain, even using laborious manual methods. Additionally, there are specific limitations about the cohort itself. Inevitably, using a secondary-care tissue-first selection process introduces inherent spectrum bias. This is a strength in terms of outcome enrichment but means that SteatoSITE will have less value for modeling the population-level natural history of MASLD. Indeed, patient characteristics (higher age and body mass index (BMI)), more frequent comorbidities and baseline disease severity likely explain the higher incidence of outcomes in SteatoSITE compared with other published MASLD cohorts^11,61,62^. The demographic makeup of the SteatoSITE cohort also lacks ethnic diversity (majority white Scottish), so caution is advised about the generalizability of findings to other geographical areas and ethnic populations. Finally, confounding due to unsuspected^63^ or poorly recorded alcohol use is a perennial issue in SLD research. To exclude erroneous cases of alcohol-related liver disease in SteatoSITE, we manually reviewed every individual clinical history section of the pathology request forms and each subsequent diagnostic report from the biorepository databases and carefully checked the cause-of-death data. We also collected International Classification of Diseases (ICD)-10 codes for alcohol-use disorders as indicated by consensus guidelines^21^. However, data on alcohol consumption (in grams per week) and drinking patterns are limited by this study’s retrospective routine/real-world nature and the lack of detailed and standardized recording of alcohol-related data in historical EHRs. Yet even in prospective studies, alcohol is a likely confounder, as highlighted by the measurement of ethyl glucuronide in hair that detected harmful alcohol consumption in 29% of patients with ‘presumed NAFLD’^63^. SteatoSITE has laid the groundwork for a variety of important research themes in MASLD, providing a rich multimodal database to inform drug and biomarker development^64^ and a catalog of high-resolution whole-slide images as a valuable testbed for novel digital pathology algorithms. Here we describe SteatoSITE v.1, but our intention is that this resource will be added to and refined, following further research and evolving disease classifications, to enhance its content and operability.

## Methods

### Regulatory approvals

Anonymized tissue was supplied after approval by the National Health Service Research Scotland (NRS) Biorepository Network (Reference: SR1032; 2 August 2018). Unified transparent approval for unconsented data inclusion in this pan-Scotland project was provided by the West of Scotland Research Ethics Committee 4 (Reference: 20/WS/0002; 18 February 2020), Public Benefit and Privacy Panel for Health and Social Care (PBPP; Reference: 1819-0091; 4 June 2021), Institutional Research & Development departments and Caldicott Guardians.

### Study cohort and sample collection

Initial case selection was on the basis of the availability of archival liver tissue (from biopsies, resections or explants that were surplus to diagnosis) in FFPE blocks available in the NRS Biorepository Network, with the clinical and/or histological diagnosis of NAFLD (MASLD) and meeting the following inclusion/exclusion criteria:

(1) Inclusion criteria—men or women; >18 years of age at the time of tissue sampling; all ethnic groups, socioeconomic backgrounds and health status; dead or alive at the time of inclusion into the data commons.

(2) Exclusion criteria—cases were excluded if any of the following applied: documented history of chronic liver disease of any non-MASLD etiology, including alcohol-related liver disease, chronic viral hepatitis, hemochromatosis, Wilson’s disease, autoimmune hepatitis, primary biliary cholangitis, or primary sclerosing cholangitis, and patients with excessive alcohol use documented in the clinical data supplied on the specimen request form (>21 units per week for men, >14 units per week for women) or histological features indicating a secondary non-MASLD diagnosis.

FFPE blocks of normal non-lesional liver from 39 control cases, defined as liver samples without fat deposition or any active primary parenchymal disease, were identified from resection specimens.

### Clinical data extraction and processing

After approval from the NHS Scotland PBPP, we obtained relevant unconsented clinical data from three of the four regional Safe Havens that cover 12 of the 14 territorial health boards in Scotland, including demographics (age, gender, ethnicity, Scottish Index of Multiple Deprivation (SIMD)), diagnostic coding (ICD-9, ICD-10) for inpatient and outpatient episodes and procedures (OPCS Classification of Interventions and Procedures (OPCS-4)), routine laboratory blood tests (biochemistry and hematology), liver transient elastography (FibroScan) where available, prescribing information and cancer and death registry data. The comprehensive list of variables (including ICD codes) is shown in Supplementary Table 1. To maximize future comparability and generalizability of results across studies, we followed recent expert consensus guidelines for using administrative coding in EHR-based research of MASLD^21^.

The EHR data was uploaded to the Precision Medicine Scotland-Innovation Centre (PMS-IC) Healthcare Landing Zone from the NHS Safe Havens as comma-separated value (.csv) files. In areas where the Safe Havens had poor coverage from their national or regional datasets, further data files were provided by the Biorepository Network. In total, 38 .csv files containing 1,450 columns and 1,055,618 rows were supplied for processing. On upload, data was quality controlled, processed and further minimized to comply with regulatory requirements and ethical approvals.

A Python (v.3.9) script was developed to carry out these data-processing tasks. These included data cleaning, data restructuring and the standardization of file inputs to resolve differences in file encoding, text delimitation and formatting. Where appropriate, differences in data labeling were resolved by adopting the naming conventions used by the NHS Greater Glasgow and Clyde Safe Haven. After standardizing case and spacing, 962 distinct columns remained from the original 1,450 columns. After a more detailed review, columns containing overlapping clinical information were relabeled and merged. This further reduced the dataset to 534 distinct columns, which are present in the final project database.

A final key requirement of the pseudonymization processing was to ensure that all event dates were obfuscated to minimize reidentification risk while allowing for clinically meaningful patient timelines to be established. To achieve this, the duration of time between the specimen acquisition date and the clinical event recorded in the EHR was calculated as Δ*t* in seconds and stored.

### Histopathology methods

Blocks for which sufficient tissue was available were assigned a study ID, and samples were cut for histology (3 × 4 µm) and, where possible, RNA extraction (4 × 10 µm). Unstained sections were sent for histological staining by the NHS Lothian pathology laboratory and curls for RNA extraction at the Genetics Core in the Edinburgh Clinical Research Facility. Only NRS biorepository staff independent of the study group held the participant key; they passed data-linkage documents to the local NHS Safe Havens, which were also independent of the study group. No information from the original clinical pathology report was supplied with the sections.

One section from each case was H&E-stained, and one section was PSR-stained. Stained sections of control cases were reviewed to ensure that no histopathological abnormalities were present. Stained sections of MASLD-spectrum cases were scanned at ×20 depth on a Hamamatsu NanoZoomer whole-slide scanner. Whole-slide image files are available as part of the SteatoSITE resource to allow further or repeat subjective or computational analysis by users.

Raw .ndpi files of H&E- and PSR-stained MASLD-spectrum cases were uploaded to a custom server installation of OMERO^65^ hosted by PMS-IC (Glasgow, UK).

In June 2019, before SteatoSITE case scoring, three consultant pathologists and participants in the UK National Liver External Quality Assurance scheme (T.J.K., G.K., P.K.) undertook an in-person scoring-harmonization exercise during which examples of histological features scored in the NAS^18^ and SAF^66^ scoring systems and for staging using NASH-CRN and modified Ishak^67^ systems were reviewed. To assess inter-rater performance following this meeting, scans of H&E- and PSR-stained sections from 20 MASLD-spectrum cases including biopsies, explants and resections were each scored by the three pathologists. For ordinal variables, Light’s kappa (square weighted, for >2 raters) was calculated using the ‘psy’^68^ package (v.1.2), and intraclass correlation coefficient, Krippendorf’s alpha and Kendall’s W were calculated using the ‘irr’^69^ package (v.0.84.1). For the single scored categorical variable (histological diagnosis of NASH), Light’s kappa and Krippendorf’s alpha were calculated.

Each pathologist was randomly allocated one-third of the cases and scored the whole-slide images in the OMERO platform, entering the scores as key–value pairs associated with each image (NAS and SAF features for H&E, NASH-CRN and modified Ishak scores for PSR).

A duplexed classification script was applied to the raw .ndpi scans of PSR-stained sections. An initial ‘whole tissue’ classifier was applied, and small artifacts on the image were removed from the mask on the basis of size. A second pixel classifier of random trees (‘RTrees’) type with ‘gaussian’ and ‘weighted deviation’ features selected, pretrained by one pathologist (T.J.K.), was applied to classify pixels into the histological classes ‘fat’, ‘psr’ and ‘other tissue’ in the ‘whole tissue’ masked area, and the total number of pixels for each class and the percentage fat and PSR-positive pixels were returned. Whole-slide scan processing was undertaken using command-line execution of a QuPath (v.0.2.3)^70^ script on the University of Edinburgh’s Edinburgh Compute and Data Facility Linux compute cluster (Eddie). Classified images were reviewed by one pathologist (T.J.K.), independent of all other data, and cases were excluded if large amounts of liver capsule or other artifacts had been included in the classification.

### Time-to-event analysis

Analysis using clinical and histopathological data only was undertaken in R^71^ (v.4.1.0) using the packages ‘survival’ (v.3.3-1)^72^, ‘survminer’ (v.0.4.9)^73^ and ‘finalfit’ (v.1.0.5)^74^. Only needle biopsy cases were used for time-to-event analysis. Decompensation events in the clinical data extract were those defined by a combination of ICD codes and UK OPCS-4 codes identifying activity relating to cirrhosis-related hospital admissions activity^21,22^. Decompensation and HCC event analysis was only undertaken on biopsy cases for which the first decompensation or HCC-related coding was present in the clinical data extract after the biopsy date; and analysis was undertaken using death as a competing risk using Cox regression on the cause-specific hazard and Fine–Gray regression for proportional-hazards modeling of the subdistribution hazard as a recommended comprehensive approach^75^. Kaplan–Meier estimator curves of all-cause mortality or event for assigned NASH-CRN fibrosis stages were compared by log-rank testing corrected for multiple comparisons using the Benjamini and Hochberg method in ‘survminer’^72^. Clustering of survival/event curves was determined using a k-means bootstrapped method^20^. Hazard ratios and 95% confidence intervals were derived from standard Cox regression models comparing mortality and rates of new-onset decompensation events with NASH-CRN stage, age at biopsy date and gender as covariates. The assumption of proportional hazards for fitted Cox regression models was verified by examining plots of scaled Schoenfeld residuals against time for each covariate.

### Multiplex immunofluorescence

mIF staining was performed using the MultiOmyx platform according to ref. ^76^. This step was performed using a single 4 μm FFPE slide: for each staining round, two cyanine dye-labeled (Cy3, Cy5) antibodies were paired. A custom multiplex panel was created consisting of 17 markers, for a total of nine rounds of antibody staining performed in sequence on the FFPE slides. In the cases of triggering receptor expressed on myeloid cells 2 (TREM2) and CD9 antigen (CD9), which were applied as primary–secondary antibodies, samples were incubated with primary TREM2 and CD9 antibody followed by incubation with a species-specific secondary antibody conjugated to cyanine 5 or 3 (Cy5 or Cy3). The staining signal was then imaged and followed by a dye-inactivation step, enabling repeated rounds of staining. The proprietary deep learning–based workflow NeoLYTX (v.2.0) was subsequently applied to identify individual cells and perform cell classification for each marker, and the phenotype of each cell was determined through coexpression analysis.

Antibodies for MultiOmyx analysis, by staining order, were rabbit anti-TREM2 (polyclonal, ProteinTech, Catalog no. 13483-1-AP, Vendor Lot ID NG) mouse anti-MNDA (253A, Abcam, Catalog no. ab270556, Vendor Lot ID GR3326911), rabbit anti-CD9 (EPR2949, Abcam, Catalog no. ab195422, Vendor Lot ID GR3282696), mouse anti-CD66b (G10F5, BioLegend, Catalog no. 93231, Vendor Lot ID B276347), mouse anti-CD11B (238439, R&D Systems, Catalog no. MAB16992, Vendor Lot ID KGZ0418101), rabbit anti-DC-SIGN (D7F5C, Cell Signaling Technology, Catalog no. 13193, Vendor Lot ID 2), rabbit anti-Ki67 (SP6, Abcam, Catalog no. ab231172, Vendor Lot ID GR3277378), rabbit anti-IDO1 (SP260, Abcam, Catalog no. ab228468, Vendor Lot ID GR3208566), rabbit anti-CD11c (D3V1E, Cell Signaling Technology, Catalog no. 45581BF, Vendor Lot ID 2), rabbit anti-PD-L1 (SP142, Abcam, Catalog no. ab236238, Vendor Lot ID GR3246745), rabbit anti-CD14 (EPR3652, Abcam, Catalog no. ab209971, Vendor Lot ID GR316076), mouse anti-CD16 (DJ130c, Thermo Fisher Scientific, Catalog no. MA1-84008, Vendor Lot ID TK2673378), mouse anti-CD68 (KP1, BioLegend, Catalog no. 98998, Vendor Lot ID B297229), mouse anti-CD163 (EDHu-1, Bio-Rad, Catalog no. MCA1853, Vendor Lot ID 149022A), mouse anti-HLA DQ/DR/DP (WR18, Novus Biologicals, Catalog no. NB100-64358, Vendor Lot ID 1808), mouse anti-CD33 (44M12D3, Novus Biologicals Catalog no. NBP2-22377, Vendor Lot ID 1127455612D3) and mouse anti-SMA (1A4, Sigma-Aldrich, Catalog no. A5228, Vendor Lot ID 037M4805V).

### RNA-seq methods

RNA extraction was performed by the Genetics Core (Clinical Research Facility, Western General Hospital, Edinburgh, UK) from 4 × 10 µm curls of FFPE archival human NAFLD liver, using the Qiagen miRNeasy FFPE Kit according to the manufacturer’s instructions. Quality control was by Qubit to measure RNA yield (and any potential DNA contamination) and Agilent Bioanalyzer to assess DV_200_. Samples with DV_200_ below 30% were not progressed for sequencing.

Libraries were prepared at PMS-IC using the low-input Takara Bio SMARTer Stranded Total RNA-Seq Kit v.2 (Pico Input Mammalian).

Sequencing data were generated by Edinburgh Genomics (University of Edinburgh, UK) using the Illumina NovaSeq 6000 platform. Libraries were sequenced over a total of 22 S2 flow cells. Reads were trimmed using ‘Cutadapt’ (v.cutadapt-1.9.dev2)^77^ and aligned to the reference genome (GRCh38) using ‘STAR’ (v.2.5.2b)^78^. Reads were assigned to features using ‘featureCounts3’ (v.1.5.1)^79^ with a .gtf file from Ensembl (annotation v.84).

For further analyses, R (v.4.1.2) was used. Samples with fewer than one million counts or 70% mapped reads were removed. Any specimen found to include HCC on histological review was excluded from further RNA-seq analyses.

Reads were normalized using the weighted trimmed mean of *M* values method^80^. Differential analysis was carried out with ‘limma-voom’ (v.3.28.14) with the protein-coding genes. Statistical significance of genes was determined by an adjusted *P* value according to the Benjamini–Hochberg procedure of *P* < 0.05 and absolute FC ≥1 (ref. ^81^).

**Extended Data Fig. 9:**
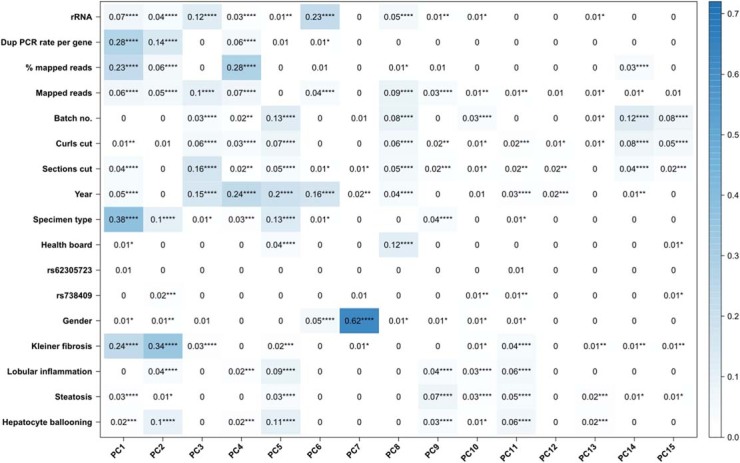
Correlations between the main principal components and the clinical data. *P*-values of Spearman correlation test. * *P* < 0.05, ** *P* < 0.01, ** *P* < 0.001, **** *P* < 0.0001.

PCA was performed to identify covariates that significantly correlated with the main principal components so they could be controlled for downstream analyses (Extended Data Fig. 9). For this reason, gender was included as an additive effect in the linear model used for differential expression when comparing fibrotic stages and NAS score. GSEA^82^ was performed with ‘clusterProfiler’ (v.4.0.5)^83^. Data were visualized with ‘ggplot2’ (v.3.3.5)^84^ and ‘clusterProfiler’.

For genes *PNPLA3*, *HSD17B13*, *TM6SF2* and *MBOAT7* containing prespecified variants (rs738409, rs72613567, rs58542926 and rs641738, respectively), the Genome Analysis Toolkit (v.4.0.1.2)^85^ was used to call genotypes at all sites. The ‘HaplotypeCaller’ tool from the Genome Analysis Toolkit was used to produce a single genomic variant call format (GVCF) file per sample, with the following parameters: ‘--standard-min-confidence-threshold-for-calling 20’; ‘-dont-use-soft-clipped-bases’; ‘-L genes.bed’; ‘-ERC GVCF’, where ‘genes.bed’ is a bed file containing the genome coordinates of the genes of interest (padded by 1 KB in both directions to cover upstream and downstream SNPs). The ‘GenotypeGVCFs’ tool was then run with the parameter ‘--include-non-variant-sites’ to produce a single VCF file per sample. ‘SnpEff’ was used to functionally annotate the variants in the VCF files.

The Multi-subject Single-cell Deconvolution tool (v.0.1.1)^86^ was run using R (v.3.6.3) to compare the counts from each bulk RNA-seq sample to reference scRNA-seq data from five healthy and five cirrhotic livers^30^ to estimate the proportions of each subtype of various lineages of non-parenchymal cells. This was done separately for each of the macrophage (non-circulating), B cells, mesenchyme, endothelial and T-cell lineages. In each analysis, only the scRNA-seq data from cells annotated as belonging to the lineage were used as a reference in the deconvolution, so the sum of estimated proportions of subtypes in each lineage equals one.

To assess the association between histological scores and proportions of various cell subtypes, the Spearman’s partial rank correlation coefficient was calculated using the R package ‘ppcor’ (v.1.1) between the proportion of each subtype and the histological score, accounting for age and gender. Gender was converted to a numerical value by setting ‘male’ to ‘1’ and ‘female’ to ‘2’. The histological data contained the ‘fibrosis stage’, which includes scores ‘1a’, ‘1b’ and ‘1c’, with ‘1a’ being the least severe and ‘1c’ being the most severe. To perform a rank correlation, these scores were converted to 1, 1.25 and 1.5, respectively. The association between proportion of each subtype and patient outcome (all-cause mortality or a decompensation event) was also assessed using Pearson’s correlation. For any given timepoint, samples were given a ‘0’ if the event had not occurred or a ‘2’ if the event had occurred and were excluded if the patient was censored at that time.

Samples with fewer than one million reads assigned to genes in the bulk RNA-seq analysis, that lacked a patient age or that did not come from a biopsy were omitted.

DEGs between F3/F4 and F0/F1 biopsy cases were identified with ‘limma-voom’ in R. The criteria for statistical significance were adjusted *P* < 0.05 and absolute FC ≥ 1. Univariate Cox analysis was carried out to determine the DEGs associated with decompensation events according to an adjusted *P* < 0.01. Next, lasso-penalized Cox regression was used to filter features using ‘glmnet’ ${\sum }_{i=1}^{n}{{\text{expression}}}_{i} \times {{\text{coefficient}}}_{i}$. Patients were divided into high- and low-risk groups according to the median score. Kaplan–Meier analysis was performed using ‘survival’ (v.3.4-0) and ‘survminer’ (v.0.4.9) packages. A time-dependent ROC curve was created with the ‘timeROC’ package (v.0.4) to evaluate the predictive ability of the signature.

Transcriptional network inference and regulon analysis was undertaken in R (v.4.1.0) using the ‘RTN’ package (v.2.16.0)^87^. Normalized gene expression of all sequenced SteatoSITE samples was used to infer regulons corresponding to all TFs compiled by ref. ^33^ by permutation analysis with the non-parametric estimator of mutual information and a *P* value cut-off of 1.75 × 10^−7^ to correct for multiple hypothesis testing. Unstable gene–TF interactions were removed by bootstrap analysis to produce a consensus network. The ARACNe algorithm^88^ using the data-processing inequality theorem to enrich regulons with direct TF–gene target interactions was used to remove the weakest interaction in any triplet composed of two TFs with a common gene target; the threshold for filtering was determined from the null distribution derived in the permutation and bootstrapping steps.

To determine regulon activity scores for each sample, the tni.gsea2() function was used to calculate differential expression of each gene relative to the expression in the entire cohort, and the ranked list of genes was used to form a differential expression signature that was used alongside the TRN to determine differential sample-specific regulon activity.

Regulon activity profiles across disease stage were placed into four clusters by unsupervised soft clustering using the ‘Mfuzz’ package (v.2.52.0). The fuzzifier parameter, ‘m’, was directly estimated from the data using mestimate() to apply the method of ref. ^89^.

The ‘RTNsurvival’ package (v.1.16.0)^90^ was used to undertake time-to-event analysis with *THRB* regulon activity. The cutpoint for *THRB* regulon activity was calculated using surv_cutpoint() from the ‘survminer’ package, applying maximally selected rank statistics of the ‘maxstat’ package (v.0.7-25) with a minimum proportion of 0.25.

We developed an open-access gene browser on the basis of the START app^91^, which will ultimately be hosted on the SteatoSITE website (https://steatosite.com/). The ‘shiny’ R package allows the production of interactive plots and charts and is already widely used to allow users to explore RNA-seq data and create their own customized figures. A ‘shiny’ interface was created to allow scientists to visualize the results of the analysis of differential gene expression according to NASH-CRN fibrosis stage.

### Inclusion and ethics statement

SteatoSITE was a consortium project involving the University of Edinburgh, University of Glasgow, NHS Greater Glasgow and Clyde and Eagle Genomics; roles and responsibilities were agreed on among collaborators ahead of the research. The study has included local researchers throughout the research process: study design, study implementation, data ownership, intellectual property and authorship.

### Reporting summary

Further information on the research design is available in the Nature Portfolio Reporting Summary linked to this article.

## Online content

Any methods, additional references, Nature Portfolio reporting summaries, source data, extended data, supplementary information, acknowledgements, peer review information; details of author contributions and competing interests; and statements of data and code availability are available at 10.1038/s41591-023-02602-2.

### Supplementary information

Supplementary Information Supplementary Tables 1–11.

Reporting Summary

## Data Availability

Hepatic bulk RNA-seq data are deposited in the European Nucleotide Archive (https://www.ebi.ac.uk/ena; study accession number: PRJEB58625). Gene expression data are also freely available for user-friendly interactive browsing online at https://shiny.igc.ed.ac.uk/SteatoSITE_gene_explorer/. SteatoSITE has delegated ethics from West of Scotland Research Ethics Committee 4 (Reference: 20/WS/0002; 18 February 2020) allowing the granting of access to the full dataset (histopathology scoring, hepatic bulk RNA-seq data, EHR data) only in the PMS-IC secure environment to third parties by application (full details at https://steatosite.com/researchers/), overseen and reviewed by the SteatoSITE Scientific Advisory Board.

## Code availability

The codes used for the data analyses in this study can be made available by contacting the corresponding author. Access to codes will be granted for requests for academic use within four weeks of application. R is required software to use the code.
