# Supplementary material for: An integrated gene-to-outcome multimodal database for metabolic dysfunction-associated steatotic liver disease
Source: Nat Med. 2023 Oct 30;29(11):2939–53. doi: 10.1038/s41591-023-02602-2 (PMC10667096; doi:10.1038/s41591-023-02602-2)
Supplement: Supplementary file 1 — Supplementary Tables 1–11. [file 41591_2023_2602_MOESM1_ESM.pdf]

nature medicine

Resource

https://doi.org/10.1038/s41591-023-02602-2

An integrated gene-to-outcome multimodal database for metabolic dysfunction-associated steatotic liver disease

In the format provided by the authors and unedited

Supplementary Table 1

Description(s)	Code(s) - ICD, OPCS, BNF
ICD09/ICD10 disease and health related problems; any applied as diagnosis or procedure and the date	
Type 2 diabetes	250.00 (9) E11.9 (10)
Hypertension, hypertensive heart disease, hypertensive kidney disease	401.0, 401.1, 401.9 402.01 402.11 402.91 402.00 402.10 402.90 403.00 403.01 403.10 403.11 403.90 403.91 (9) I10 I11.0 I11.9 I12.0 I12.9 (10)
Ischaemic Heart disease (acute, chronic)	411.89 414.8 414.9 (9) I24.9 I25.9 (10)
Angina	411.1 413.9 (9) I20.0 I20.09 (10)
MI	410 414.2 (9) I21.9 I25.82 (10)
Dyslipidaemia/hyperchesterolaemia	272.2 272.0 (9) E78.5 E78.00 E78.01 (10)
Chronic Kidney disease	585.9 (9) N18.9 (10)
Ascites	789.5 (9) R18.8 (10)
encephalopathy	348.30 (9) G93.40 (10)
hepatocellular carcincoma	155.0 (9) c22.0 (10)
NAFLD diagnosis	571.8 (9) K75.81, K76.0 (10)
Obesity	278.00 (9) E66.9 (10)
Cirrhosis	571.5 (9) K74.60 (10)
Diabetic nephropathology	250.40 (9) E11.29 (10)
Intermittent claudication	443.9 (9) l70.213 (10)
Varices (bleeding, without bleeding)	456.1, 456.0 (9) I85.00, I85.01 (10)
Hepatic encelphalopathy	572.2 (9) K72.90 (10)
Spontaneous bacterial peritonitis	567.23 (9) K65.2 (10)
Hepatorenal syndrome	572.4 (9) k76.7 (10)
Hepatic failure	572.2/572.4/573.3/573.8/572.8 (9) K72.90 (10)

Description(s)	Code(s) - ICD, OPCS, BNF
NASH	571.8 (9) k75.81 (10)
Fatty (change of) liver, not elsewhere classified	K76.0
Liver disease, unspecified	573.9 (9) k76.9 (10)
Hepatitis fibrosis	571.5 (9) k74.0 (10)
Hepatic sclerosis	571.5 (9) k74.1 (10)
Hepatic fibrosis with hepatic sclerosis	571.5 (9) k74.2 (10)
Aany other unspecified cirrhosis of the liver	571.5 (9) k74.6 (10)
Hepatomegaly not elsewhere classified	789.1 (9) R16.0 (10)
Splenomegaly not elsewher classified	789.2 (9) R16.1 (10)
Hepatomegaly with splenomegaly not elsewhere classified	R16.2 (10)
Haematemesis	578.0 (9) K92.0 (10)
Melaena	777.3 (9) K92.1 (10)
Gastrointestinal haemorrhage, unspecified	578.9 (9) K92.2 (10)
Portal hypertension	572.3 (9) k76.6 (10)
Hepatopulmonary syndrome	573.5 (9) k76.81 (10)
Acute kidney injury	584.9 (9) N17.9 (10)
Hepatocellular carcinoma	155.0 (9) C22.0 (10)
Cholangiocarcinoma	155.1 (9) C22.1 (10)
Malignant neoplasms of digestive organs	C15-C26 (10)
Malignant neoplasms of female genital organs	C51-C58 (10)

Description(s)	Code(s) - ICD, OPCS, BNF
Obstructive sleep apnoea	327.23 (9) G47.33 (10)
Blood_Biochemistry (SCI_store): all per patient, over the range provided with specific dates	
Alanine aminotransferase (ALT)	NA
Aspartate aminotransferase (AST)	NA
Bilirubin	NA
Alkaline phosphatase (ALP)	NA
Gamma-glutamyl transpeptidase (GGT)	NA
Sodium	NA
Potassium	NA
Urea	NA
Creatinine	NA
Estimated Glomerular Filtration Rate (eGFR)	NA
Albumin	NA
Lipids (are these covered by Triglycerides, HDL, LDL, VLDL?)	NA
Triglycerides	NA
HDL	NA
LDL	NA
Glucose	NA
HbA1C	NA
Haemoglobin	NA
Platelets	NA
Neutrophils 10^9/L	NA

Description(s)	Code(s) - ICD, OPCS, BNF
Lymphocytes 10^9/L	NA
Neutrophil:lymphocyte ratio (NLR)	NA
Hyaluronic acid	NA
Prothrombin time and INR	NA
HOMA-IR	NA
OPCS_04 Intervensions; when these codes from TRAK used with specific dates	
Imaging scans	U01, U08, U10, U11, U16, U17, U19, U20, U21, U36
Liver procedures (biopsy, locoregional HCC treatments, TIPSS, liver resection and transplants etc)	J01, J02, J03, J06, J10, J11, J12, J13, J14, J15, J17, J72
Endoscopy procedures	G10, GG14, G15, G16, G42, G43, G44, G45, G46, G47, H52
Paracentesis	T46
Cardiovascular procedures	K40, K41, K42, K43, K44, K45, K46, K47, K48, K49, K50, K51, K60, K61, K63, K65, K72, K75, L29, L31, L59, L63, L66, L68, L71, K74, K76
renal transplant	M01
compensation for renal failure	X40, X41, X42
compensation for liver failure	X43
Fibroscan	manual data collection
Medications: Name of medication from BNF with the dates precribed	
PPIs; H2-receptor antagonists, laxatives	1.1, 1.3, 1.6
all cardiovascular drugs: anti-hypertensives; diruretics; lipid lowering; anticoagulants	2
anti-obesity drugs	4.5
antibiotics; antibacterial	5

Description(s)	Code(s) - ICD, OPCS, BNF
Treatment for diabetes - anti-diabetic drugs, insulin	6.1
Chemotherapy; Immunosuppressants incl corticosteroids	8
Vitamins	9.6
SMR-06: Cancer; additional requirements when any of the ICD-O codes with C22 (ICD-10 site): Tumour site and type codes, and ENCR incidence date	
ICD-O-2:	8170/3, 8180/3, 8160/3, 8162/3
ICD-O-3	8170/3, 8173/3, 8174/3, 8175/3, 8180/3, 8160/3, 8162/3

Supplementary Table 2

	NASH-CRN fibrosis stage				
**Variable**	**0**	**1**	**2**	**3**	**4**
Liver test, per case temporal range, median days (IQR)					
ALT	2830 (2320)	2759 (2542.5)	3051 (2793.5)	2984 (3065.75)	2379 (2456.75)
AST	1251 (2172)	1316.5 (2540)	1441 (1604.75)	1522 (2484)	856.5 (2134)
ALP	2958 (2331.5)	2898 (2691.75)	3168 (2820.5)	3143 (3146.75)	2580 (2701.5)
Bilirubin	2960 (2477)	2780.5 (2696)	3380 (3100.25)	3096 (3641.75)	2444 (2560)
INR	569 (1969.25)	827 (2380)	816.5 (2344)	1291.5 (2442.75)	1619.5 (2152.25)
Prothrombin time	888 (2253)	1221 (2542.5)	942.5 (2543.75)	1501.5 (2596.75)	1921 (2252.75)
Other laboratory results, per case temporal range, median days (IQR)					
Creatinine	2760.5 (2146)	2417 (2598)	2670 (2630.5)	2791 (2437)	2314 (2671)
Albumin	2297 (2723.5)	2404 (2992.5)	2662 (2745)	2772 (3239.5)	2301 (2640.75)
Platelets	2913 (2332)	2934 (2684)	3339 (2627.5)	3143 (2998)	2681.5 (2648.25)

Supplementary Table 3

	**Number of cases (%)**
Biopsy cases	
Diagnosis (deranged LFTs)	428 (45.5)
Staging/diagnosis (deranged LFTs)	197 (21)
Unknown	18 (1.9)
Staging	16 (1.7)
Resection cases	
Colorectal carcinoma metastasis	150 (16)
Hepatocellular carcinoma	38 (4)
Benign, not otherwise specified	12 (1.3)
Hepatic adenoma	6 (0.6)
Focal nodular hyperplasia	5 (0.5)
Biliary tract carcinoma	4 (0.4)
neuroendocrine tumour	4 (0.4)
Non colorectal metastasis	2 (0.2)
Unknown	1 (0.1)
Bile duct adenoma	1 (0.1)
Completion excision of gallbladder bed	1 (0.1)
Mucinous cystic neoplasm	1 (0.1)

Supplementary Table 4

Histological feature	Light's kappa	Intraclass correlation coefficient	Krippendorf's alpha	Kendall's W
NAS ballooning	0.620	0.627	0.621	0.754
SAF ballooning	0.374	0.392	0.334	0.592
NAS inflammation	0.251	0.303	0.176	0.549
SAF inflammation	0.131	0.165	0.107	0.466
NAS/SAF steatosis	0.826	0.830	0.823	0.887
Modified Ishak fibrosis stage	0.954	0.958	0.945	0.967
NASH-CRN fibrosis stage	0.923	0.934	0.912	0.947
Histological diagnosis of NASH	0.416	NA	0.408	NA

Supplementary Table 5

All-cause mortality			
	all	HR (univariable)	HR (multivariable)
Age at biopsy			
Mean (SD)	52.1 (13.5)	1.08 (1.06-1.10, p<0.001)	1.07 (1.05-1.10, p<0.001)
Gender, number (%)			
F	312 (47.3)	-	-
M	347 (52.7)	0.60 (0.41-0.87, p=0.008)	1.11 (0.74-1.65, p=0.617)
NASH-CRN fibrosis stage, number (%)			
0	198 (30.0)	-	-
1	150 (22.8)	0.92 (0.48-1.79, p=0.816)	0.85 (0.44-1.66, p=0.634)
2	100 (15.2)	1.69 (0.89-3.19, p=0.108)	1.07 (0.56-2.06, p=0.837)
3	115 (17.5)	2.54 (1.42-4.53, p=0.002)	1.78 (0.99-3.21, p=0.056)
4	96 (14.6)	5.32 (3.12-9.09, p<0.001)	2.79 (1.60-4.87, p<0.001)

Number in dataframe = 659, Number in model = 659, Missing = 0, Number of events = 112, Concordance = 0.782 (SE = 0.020), R-squared = 0.146 (Max possible = 0.854), Likelihood ratio test = 103.888 (df = 6, p = 0.000)

Supplementary Table 6

Biopsy cases for all-cause mortality modelling						
**NASH-CRN fibrosis stage**	**Status (0, alive; 1, dead)**	**Gender**	**RNAseq success**	**Post-biopsy period to death/censoring (median days ± IQR)**	**Age at biopsy (years ± SD)**	**Number of cases (percent)**
0	0	F	No	3969 ± 3641.25	48.64 ± 13.52	14 (2.12)
0	0	F	Yes	2586 ± 3111	51.61 ± 12.21	61 (9.26)
0	0	M	No	2968 ± 3497.25	38.23 ± 11.36	22 (3.34)
0	0	M	Yes	2495 ± 2859	43.68 ± 12.08	77 (11.68)
0	1	F	No	130 ± 96.5	67 ± 4.36	3 (0.46)
0	1	F	Yes	2319.5 ± 4307.75	57.4 ± 10.43	10 (1.52)
0	1	M	No	3843 ± 3826	54.8 ± 19.49	5 (0.76)
0	1	M	Yes	1987.5 ± 1993.5	62 ± 10.79	6 (0.91)
1	0	F	No	2940.5 ± 1320.75	55.61 ± 10.67	18 (2.73)
1	0	F	Yes	1556 ± 1522.75	52.04 ± 12.35	28 (4.25)
1	0	M	No	2617 ± 1944.75	40.12 ± 15.53	16 (2.43)
1	0	M	Yes	2379 ± 2869	46.6 ± 12.87	73 (11.08)
1	1	F	No	2532 ± 0	59 ± NA	1 (0.15)
1	1	F	Yes	1686 ± 2919	61.7 ± 8.43	10 (1.52)
1	1	M	Yes	4465 ± 2358.75	52 ± 14.17	4 (0.61)

Biopsy cases for all-cause mortality modelling						
**NASH-CRN fibrosis stage**	**Status (0, alive; 1, dead)**	**Gender**	**RNAseq success**	**Post-biopsy period to death/censoring (median days ± IQR)**	**Age at biopsy (years ± SD)**	**Number of cases (percent)**
2	0	F	No	2734.5 ± 1019.5	67.5 ± 12.02	2 (0.3)
2	0	F	Yes	2417 ± 3130	54.83 ± 12.11	29 (4.4)
2	0	M	No	3570 ± 1358.25	44 ± 10.35	10 (1.52)
2	0	M	Yes	1889 ± 1645.5	49.84 ± 14.16	43 (6.53)
2	1	F	No	3048.5 ± 2208.5	62 ± 1.41	2 (0.3)
2	1	F	Yes	647.5 ± 3021.75	68.12 ± 11.29	8 (1.21)
2	1	M	Yes	1511 ± 394.5	60.33 ± 9.42	6 (0.91)
3	0	F	No	3316 ± 3348.75	53.38 ± 10.72	8 (1.21)
3	0	F	Yes	1766 ± 1611	56.55 ± 12.13	49 (7.44)
3	0	M	No	3639 ± 1098	48.4 ± 14.17	5 (0.76)
3	0	M	Yes	1660 ± 1326	55.13 ± 10.67	30 (4.55)
3	1	F	No	2142 ± 2517.5	55.67 ± 10.21	3 (0.46)
3	1	F	Yes	2293.5 ± 2033	62.2 ± 10.36	10 (1.52)
3	1	M	No	100 ± 0	72 ± NA	1 (0.15)
3	1	M	Yes	2341 ± 1680	59.44 ± 14.48	9 (1.37)

Biopsy cases for all-cause mortality modelling						
**NASH-CRN fibrosis stage**	**Status (0, alive; 1, dead)**	**Gender**	**RNAseq success**	**Post-biopsy period to death/censoring (median days ± IQR)**	**Age at biopsy (years ± SD)**	**Number of cases (percent)**
4	0	F	No	2142.5 ± 760.5	59 ± 10.1	4 (0.61)
4	0	F	Yes	1934 ± 1370	60.39 ± 11.26	33 (5.01)
4	0	M	No	2556 ± 972.5	59.67 ± 10.12	3 (0.46)
4	0	M	Yes	1146.5 ± 1500.25	52.27 ± 10.43	22 (3.34)
4	1	F	No	1845 ± 1082	60.83 ± 10.23	6 (0.91)
4	1	F	Yes	861 ± 1945	63.15 ± 7.5	13 (1.97)
4	1	M	No	1774.5 ± 1755.75	61.25 ± 5.44	4 (0.61)
4	1	M	Yes	1113 ± 1104	59 ± 10.94	11 (1.67)

Supplementary Table 7

All-cause mortality, NAS scoring			
	**all**	**HR (univariable)**	**HR (multivariable)**
NASH-CRN fibrosis, number (%)			
0	198 (30.0)	-	-
1	150 (22.8)	0.92 (0.48-1.79, p=0.816)	1.10 (0.56-2.16, p=0.783)
2	100 (15.2)	1.69 (0.89-3.19, p=0.108)	1.89 (0.95-3.74, p=0.069)
3	115 (17.5)	2.54 (1.42-4.53, p=0.002)	3.20 (1.64-6.24, p=0.001)
4	96 (14.6)	5.32 (3.12-9.09, p<0.001)	5.65 (3.12-10.24, p<0.001)
NAS lobular inflammation, number (%)			
0	94 (14.3)	-	-
1	305 (46.3)	1.14 (0.67-1.94, p=0.623)	0.96 (0.52-1.76, p=0.896)
2	220 (33.4)	1.27 (0.72-2.25, p=0.412)	1.03 (0.49-2.16, p=0.941)
3	40 (6.1)	1.45 (0.57-3.68, p=0.434)	1.36 (0.47-3.94, p=0.569)
NAS steatosis, number (%)			
0	22 (3.3)	-	-
1	242 (36.7)	0.90 (0.39-2.10, p=0.808)	1.12 (0.44-2.83, p=0.812)
2	245 (37.2)	0.67 (0.28-1.58, p=0.362)	0.83 (0.31-2.26, p=0.721)
3	150 (22.8)	0.37 (0.14-0.96, p=0.040)	0.47 (0.16-1.38, p=0.169)
NAS hepatocyte ballooning, number (%)			
0	248 (37.6)	-	-
1	218 (33.1)	1.23 (0.79-1.91, p=0.358)	1.33 (0.70-2.52, p=0.381)
2	193 (29.3)	1.47 (0.91-2.36, p=0.117)	1.22 (0.56-2.66, p=0.621)

Number in dataframe = 659, Number in model = 659, Missing = 0, Number of events = 112, Concordance = 0.699 (SE = 0.028), R-squared = 0.085 (Max possible = 0.854), Likelihood ratio test = 58.506 (df = 13, p = 0.000)

All-cause mortality, NAS scoring			
	**all**	**HR (univariable)**	**HR (multivariable)**
Histological diagnosis of NASH, number (%)			
no	305 (46.3)	-	-
yes	354 (53.7)	1.24 (0.85-1.82, p=0.270)	0.68 (0.36-1.29, p=0.240)

Number in dataframe = 659, Number in model = 659, Missing = 0, Number of events = 112, Concordance = 0.699 (SE = 0.028), R-squared = 0.085 (Max possible = 0.854), Likelihood ratio test = 58.506 (df = 13, p = 0.000)

Supplementary Table 8

Hepatic decompensation events			
	all	**Cause-specific HR (multivariable)**[1]	**Subdistribution HR (multivariable)**[2]
Age at biopsy			
Mean (SD)	51.2 (13.7)	1.01 (0.99-1.03, p=0.315)	1.00 (0.99-1.02, p=0.711)
Gender, number (%)			
F	256 (44.0)	-	-
M	326 (56.0)	0.69 (0.46-1.05, p=0.080)	0.64 (0.42-0.97, p=0.037)
NASH-CRN fibrosis stage, number (%)			
0	188 (32.3)	-	NA
1	136 (23.4)	1.08 (0.55-2.12, p=0.815)	1.09 (0.56-2.11, p=0.804)
2	91 (15.6)	1.35 (0.66-2.76, p=0.415)	1.32 (0.65-2.71, p=0.442)
3	98 (16.8)	2.63 (1.42-4.87, p=0.002)	2.54 (1.35-4.76, p=0.004)
4	69 (11.9)	6.99 (3.87-12.61, p<0.001)	6.86 (3.71-12.68, p<0.001)

[1] Concordance = 0.741 (SE = 0.025), Likelihood ratio test = 74.546 (df = 6, p = <0.0001)

[2] Concordance = 0.733 (SE = 0.026), Likelihood ratio test = 70.54 (df = 6, p = <0.0001)

Number in dataframe = 582, Number in model = 582, Missing = 0, Number of events = 106

Supplementary Table 9

Biopsy cases for hepatic decompensation modelling						
**NASH-CRN fibrosis stage**	**Decompensation status**	**Gender**	**RNAseq success**	**Post-biopsy period to event/censoring (median days ± IQR)**	**Age at biopsy (years ± SD)**	**Number of cases (percent)**
0	0	F	No	3024 ± 3366	52.46 ± 13.44	13 (2.23)
0	0	F	Yes	2586 ± 3471	51.78 ± 11.12	55 (9.45)
0	0	M	No	3128 ± 3817	39.22 ± 14.11	23 (3.95)
0	0	M	Yes	2572 ± 2904.75	44.79 ± 12.87	76 (13.06)
0	1	F	No	2715 ± 596	35.5 ± 14.85	2 (0.34)
0	1	F	Yes	834.5 ± 794.5	55.9 ± 15.59	10 (1.72)
0	1	M	No	1324.5 ± 1499.5	53.25 ± 9.91	4 (0.69)
0	1	M	Yes	370 ± 525	44.4 ± 15.19	5 (0.86)
1	0	F	No	2835 ± 1227	57.77 ± 10.98	13 (2.23)
1	0	F	Yes	1765.5 ± 1443.5	56.19 ± 12.19	26 (4.47)
1	0	M	No	2659 ± 2019	40.27 ± 16.07	15 (2.58)
1	0	M	Yes	2379 ± 3009	45.66 ± 12.43	67 (11.51)
1	1	F	No	1523 ± 2856	49.8 ± 7.92	5 (0.86)

Biopsy cases for hepatic decompensation modelling						
**NASH-CRN fibrosis stage**	**Decompensation status**	**Gender**	**RNAseq success**	**Post-biopsy period to event/censoring (median days ± IQR)**	**Age at biopsy (years ± SD)**	**Number of cases (percent)**
1	1	F	Yes	2088.5 ± 1202	52.75 ± 9.18	4 (0.69)
1	1	M	Yes	3490.5 ± 1979.75	49.5 ± 13.34	6 (1.03)
2	0	F	No	3486 ± 1771	60 ± 1.41	2 (0.34)
2	0	F	Yes	2664.5 ± 3093.25	54.75 ± 12.01	24 (4.12)
2	0	M	No	3570 ± 1358.25	44 ± 10.35	10 (1.72)
2	0	M	Yes	1833 ± 1433.5	50 ± 14.11	43 (7.39)
2	1	F	No	289 ± 0	63 ± NA	1 (0.17)
2	1	F	Yes	373 ± 1483.5	61.57 ± 16.85	7 (1.2)
2	1	M	Yes	414.5 ± 286.75	61 ± 11.22	4 (0.69)
3	0	F	No	3316 ± 3197	55.67 ± 9.97	6 (1.03)
3	0	F	Yes	1766 ± 1678	58.46 ± 12.29	41 (7.04)
3	0	M	No	2590 ± 2424.5	47.33 ± 23.59	3 (0.52)
3	0	M	Yes	1627 ± 1188	56 ± 12.75	25 (4.3)
3	1	F	No	743.5 ± 648.75	51 ± 12.52	4 (0.69)

Biopsy cases for hepatic decompensation modelling						
**NASH-CRN fibrosis stage**	**Decompensation status**	**Gender**	**RNAseq success**	**Post-biopsy period to event/censoring (median days ± IQR)**	**Age at biopsy (years ± SD)**	**Number of cases (percent)**
3	1	F	Yes	810 ± 2345	56.55 ± 13.92	11 (1.89)
3	1	M	No	244 ± 0	59 ± NA	1 (0.17)
3	1	M	Yes	1037 ± 1050.5	53.29 ± 11.97	7 (1.2)
4	0	F	No	994 ± 0	48 ± NA	1 (0.17)
4	0	F	Yes	2272 ± 1606.75	64.5 ± 7.14	12 (2.06)
4	0	M	No	1024 ± 0	65 ± NA	1 (0.17)
4	0	M	Yes	1086 ± 737.5	55.15 ± 11.22	20 (3.44)
4	1	F	No	649 ± 1179	61.67 ± 9.11	6 (1.03)
4	1	F	Yes	821 ± 1161	57.77 ± 11.83	13 (2.23)
4	1	M	No	332 ± 923.5	61 ± 8.83	4 (0.69)
4	1	M	Yes	727.5 ± 1029.25	53.5 ± 11.29	12 (2.06)

Supplementary Table 10

Hepatic decompensation events, NAS scoring			
	**all**	**HR (univariable)**	**HR (multivariable)**
NASH-CRN fibrosis, number (%)			
0	188 (32.3)	-	-
1	136 (23.4)	1.06 (0.54-2.05, p=0.873)	1.14 (0.57-2.26, p=0.717)
2	91 (15.6)	1.38 (0.68-2.82, p=0.372)	1.39 (0.65-3.00, p=0.397)
3	98 (16.8)	3.08 (1.69-5.62, p<0.001)	3.04 (1.49-6.17, p=0.002)
4	69 (11.9)	8.14 (4.67-14.17, p<0.001)	7.35 (3.87-13.96, p<0.001)
NAS lobular inflammation, number (%)			
0	85 (14.6)	-	-
1	271 (46.6)	1.30 (0.73-2.32, p=0.376)	0.82 (0.42-1.63, p=0.574)
2	194 (33.3)	1.47 (0.80-2.70, p=0.219)	0.60 (0.27-1.37, p=0.228)
3	32 (5.5)	1.03 (0.34-3.13, p=0.955)	0.48 (0.14-1.68, p=0.251)
NAS steatosis, number (%)			
0	22 (3.8)	-	-
1	212 (36.4)	0.67 (0.28-1.60, p=0.370)	0.65 (0.25-1.66, p=0.367)
2	215 (36.9)	0.68 (0.29-1.62, p=0.388)	0.65 (0.24-1.76, p=0.400)
3	133 (22.9)	0.55 (0.22-1.37, p=0.201)	0.52 (0.18-1.48, p=0.221)
NAS hepatocyte ballooning, number (%)			
0	224 (38.5)	-	-
1	191 (32.8)	1.31 (0.80-2.15, p=0.280)	1.37 (0.67-2.77, p=0.385)
2	167 (28.7)	2.72 (1.70-4.35, p<0.001)	2.14 (0.94-4.91, p=0.071)

Number in dataframe = 659, Number in model = 659, Missing = 0, Number of events = 112, Concordance = 0.699 (SE = 0.028), R-squared = 0.085 (Max possible = 0.854), Likelihood ratio test = 58.506 (df = 13, p = 0.000)

Hepatic decompensation events, NAS scoring			
	all	HR (univariable)	HR (multivariable)
Histological diagnosis of NASH, number (%)			
no	276 (47.4)	-	-
yes	306 (52.6)	1.69 (1.14-2.51, p=0.009)	0.87 (0.44-1.70, p=0.678)

Number in dataframe = 659, Number in model = 659, Missing = 0, Number of events = 112, Concordance = 0.699 (SE = 0.028), R-squared = 0.085 (Max possible = 0.854), Likelihood ratio test = 58.506 (df = 13, p = 0.000)

Supplementary Table 11

Genotype by fibrosis stage					
	**NASH-CRN fibrosis stage**				
	**0**	**1**	**2**	**3**	**4**
PNPLA3 (rs738409), number (%)					
G/G	25 (3.74)	21 (3.14)	14 (2.1)	22 (3.29)	21 (3.14)
C/G	48 (7.19)	33 (4.94)	28 (4.19)	29 (4.34)	32 (4.79)
C/C	94 (14.07)	78 (11.68)	55 (8.23)	56 (8.38)	52 (7.78)
C/T	2 (0.3)	3 (0.45)	0 (0)	1 (0.15)	0 (0)
Not called	6 (0.9)	6 (0.9)	8 (1.2)	16 (2.4)	18 (2.69)
HSD17B13 (rs72613567), number (%)					
T/T	4 (0.6)	2 (0.3)	5 (0.75)	1 (0.15)	1 (0.15)
T/TA	25 (3.74)	15 (2.25)	12 (1.8)	11 (1.65)	10 (1.5)
TA/TA	37 (5.54)	35 (5.24)	23 (3.44)	15 (2.25)	19 (2.84)
Not called	109 (16.32)	89 (13.32)	65 (9.73)	97 (14.52)	93 (13.92)
TM6SF2 (rs58542926), number (%)					
C/C	127 (19.01)	105 (15.72)	68 (10.18)	78 (11.68)	69 (10.33)
C/T	8 (1.2)	8 (1.2)	2 (0.3)	4 (0.6)	3 (0.45)
T/T	5 (0.75)	3 (0.45)	1 (0.15)	3 (0.45)	5 (0.75)
Not called	35 (5.24)	25 (3.74)	34 (5.09)	39 (5.84)	46 (6.89)
MBOAT7 (rs641738), number (%)					
C/C	8 (1.2)	6 (0.9)	6 (0.9)	10 (1.5)	10 (1.5)
T/T	10 (1.5)	17 (2.54)	11 (1.65)	11 (1.65)	23 (3.44)
T/C	0 (0)	1 (0.15)	0 (0)	2 (0.3)	1 (0.15)
Not called	157 (23.5)	117 (17.51)	88 (13.17)	101 (15.12)	89 (13.32)
