# Supplementary material for: An integrated gene-to-outcome multimodal database for metabolic dysfunction-associated steatotic liver disease
Source: Nat Med. 2023 Oct 30;29(11):2939–53. doi: 10.1038/s41591-023-02602-2 (PMC10667096; doi:10.1038/s41591-023-02602-2)
Supplement: Supplementary file 2 — Reporting Summary [file 41591_2023_2602_MOESM2_ESM.pdf]

nature portfolio

Corresponding author(s): Professor Jonathan Fallowfield

Last updated by author(s): 19/09/23

Reporting Summary

Nature Portfolio wishes to improve the reproducibility of the work that we publish. This form provides structure for consistency and transparency in reporting. For further information on Nature Portfolio policies, see our Editorial Policies and the Editorial Policy Checklist.

Statistics

For all statistical analyses, confirm that the following items are present in the figure legend, table legend, main text, or Methods section.

n/a	Confirmed	
☐	☒	The exact sample size (*n*) for each experimental group/condition, given as a discrete number and unit of measurement
☐	☒	A statement on whether measurements were taken from distinct samples or whether the same sample was measured repeatedly
☐	☒	The statistical test(s) used AND whether they are one- or two-sided<br>*Only common tests should be described solely by name; describe more complex techniques in the Methods section.*
☐	☒	A description of all covariates tested
☐	☒	A description of any assumptions or corrections, such as tests of normality and adjustment for multiple comparisons
☐	☒	A full description of the statistical parameters including central tendency (e.g. means) or other basic estimates (e.g. regression coefficient) AND variation (e.g. standard deviation) or associated estimates of uncertainty (e.g. confidence intervals)
☐	☒	For null hypothesis testing, the test statistic (e.g. *F*, *t*, *r*) with confidence intervals, effect sizes, degrees of freedom and *P* value noted<br>*Give P values as exact values whenever suitable.*
☒	☐	For Bayesian analysis, information on the choice of priors and Markov chain Monte Carlo settings
☒	☐	For hierarchical and complex designs, identification of the appropriate level for tests and full reporting of outcomes
☐	☒	Estimates of effect sizes (e.g. Cohen's *d*, Pearson's *r*), indicating how they were calculated

Our web collection on statistics for biologists contains articles on many of the points above.

Software and code

Policy information about availability of computer code

Data collection	A custom Python (version 3.9) script was developed to carry out Electronic Health Record data processing tasks.
Data analysis	When scoring the liver pathology, for ordinal variables, Light's kappa (square weighted, for >2 raters) was calculated using the 'psy' package (version 1.2), and intraclass correlation coefficient, Krippendorf's alpha, and Kendall's W were calculated using the 'irr' package (version 0.84.1) in R (version 4.1.0). Q Path open source software (version 0.2.3) was used for liver histopathological image analysis. Analysis using clinical and histopathological data only was undertaken in R (version 4.1.0) using the packages 'survival' (version 3.2-1), 'survminer' (version 0.4.9), and 'finalfit' (version 1.0.5).<br>NeoGenomics used a proprietary deep learning-based workflow NeoLYTX (version 2.0) to identify individual liver cells and perform cell classification for cell markers.<br>For RNA-seq analysis, the following software packages were used in R (version 4.1.2) : Reads were trimmed using 'Cutadapt' (version cutadapt-1.9.dev2) and aligned to the reference genome using 'STAR' (version 2.5.2b). Reads were assigned to features using 'featureCounts3' (version 1.5.1) with a igtf file from Ensembl (annotation version 84). Differential gene expression analysis was performed using limma-voom' (version 3.28.14); Gene Set Enrichment Analysis (GSEA) was performed with GSEA function from 'clusterProfiler' (version 4.0.5); data were visualized with 'ggplot2' (version 3.3.5) and 'clusterProfiler'; Cox regression was performed using 'glmnet' (version 4.1-4), and time-dependent ROC curves were created by the 'timeROC' package (version 0.4); Kaplan-Meier analysis was performed using 'survival' (version 3.4-0) and 'survminer' (version 0.4.9) packages.<br>Genome Analysis Toolkit (GATK, version 4.0.1.2) was used to call genotypes.<br>The MUlti-Subject Single Cell ('MuSiC') deconvolution tool (version 0.1.1) was run using R (version 3.6.3) for deconvolution analysis. The R package<br>`ppcor' (version 1.1) was used to assess the correlation between the proportion of hepatic cell subtypes and the histological score or clinical outcomes.

April 2023

Transcriptional network inference and regulon analysis was undertaken in R (version 4.1.0) using the 'RTN' package (version 2.16.0) implementing the ARACHNe algorithm, the 'RTNsurvival' package (version 1.16.0), 'maxstat' package (version 0.7-25), and 'Mfuzz' package (version 2.52.0).
An R Shiny app was used to develop the gene browser.
R scripts enabling the main steps of the analysis are available from the corresponding author on reasonable request.

For manuscripts utilizing custom algorithms or software that are central to the research but not yet described in published literature, software must be made available to editors and reviewers. We strongly encourage code deposition in a community repository (e.g. GitHub). See the Nature Portfolio guidelines for submitting code & software for further information.

Data

Policy information about availability of data

All manuscripts must include a data availability statement. This statement should provide the following information, where applicable:

- Accession codes, unique identifiers, or web links for publicly available datasets
- A description of any restrictions on data availability
- For clinical datasets or third party data, please ensure that the statement adheres to our policy

Hepatic bulk RNA-seq data is deposited in the European Nucleotide Archive (https://www.ebi.ac.uk/ena; study accession number: PRJEB58625). Gene expression data is also freely available for user-friendly interactive browsing online at https://shiny.igc.ed.ac.uk/SteatoSITE_gene_explorer/. SteatoSITE has delegated ethics from West of Scotland Research Ethics Committee 4 (Reference: 20/WS/0002; 18th February 2020) allowing the granting of access to the full dataset (histopathology scoring, hepatic bulk RNA-seq data, Electronic Health Record data) only within the PMS-IC secure environment to third parties by application (full details at https://steatosite.com/researchers/), overseen and reviewed by the SteatoSITE Scientific Advisory Board.

Research involving human participants, their data, or biological material

Policy information about studies with human participants or human data. See also policy information about sex, gender (identity/presentation), and sexual orientation and race, ethnicity and racism.

Reporting on sex and gender	'Gender' is reported as stated in the Electronic Health Record. Both men and women are included in the cohort. Gender was used as a covariate in analysis as there are sex-related differences in NAFLD prevalence and outcome.
Reporting on race, ethnicity, or other socially relevant groupings	Ethnicity and SIMD (Scottish Index of. Multiple Deprivation) data were collected from national administrative datasets and are reported as ethnicity and social deprivation influence NAFLD prevalence and outcome.
Population characteristics	Covariate relevant population characteristics include age (mean 55.1 years), gender (55.4% men, 44.6% women), ethnicity (White 64.5%, Asian 2.3%, unknown ethnicity 33.2%), SIMD (1 (6.9%), 2 (8%), 3 (7.8%), 4 (7.9%), 5 (8%), 6 (6.2%), 7 (5.3%), 8 (3.5%), 9 (4.4%), 10 (5.9%), unknown SIMD (36.3%)), diabetes status (32% type 2 diabetes), body mass index (median 31.3), liver fibrosis stage (F0 (n=247), F1 (n=208), F2 (n=152), F3 (n=169), F4 (n=164)), and genotypic (SNP) status (PNPLA3: GG (16.8%), GC (27.8%) and CC (54.7%).
Recruitment	This was a retrospective, observational study. Initial case selection was based on the availability of archival liver tissue (from biopsies, resections, or explants that were surplus to diagnosis) in formalin-fixed paraffin-embedded (FFPE) blocks available within the NHS Research Scotland Biorepository network, with the clinical and/or histological diagnosis of NAFLD, and meeting specific inclusion/exclusion criteria.<br>Using a secondary care tissue-first selection process introduces spectrum bias and this is acknowledged in the discussion. This is a strength in terms of outcome enrichment but means that SteatoSITE will have less value for modelling the population-level natural history of NAFLD.
Ethics oversight	Anonymised tissue was supplied after approval by the National Health Service Research Scotland (NRS) Biorepository network (Reference: SR1032; 2nd August 2018). Unified transparent approval for data inclusion in this pan-Scotland project was provided by the West of Scotland Research Ethics Committee 4 (Reference: 20/WS/0002; 18th February 2020), Public Benefit and Privacy Panel for Health and Social Care (PBPP; Reference: 1819-0091; 4th June 2021), Institutional Research & Development departments and Caldicott Guardians.

Note that full information on the approval of the study protocol must also be provided in the manuscript.

Field-specific reporting

Please select the one below that is the best fit for your research. If you are not sure, read the appropriate sections before making your selection.

☒ Life sciences ☐ Behavioural & social sciences ☐ Ecological, evolutionary & environmental sciences

For a reference copy of the document with all sections, see nature.com/documents/nr-reporting-summary-flat.pdf

Life sciences study design

All studies must disclose on these points even when the disclosure is negative.

Sample size	No sample size calculation was performed. The cohort size is a reflection of the maximum number of eligible cases across all of the Scottish

Sample size	Biorepositories at the time of data collection. To our knowledge, this is the largest collection of NAFLD cases with hepatic RNA-sequencing, digital pathology and linked clinical outcomes worldwide.
Data exclusions	There were pre-determined Quality Control criteria for RNA-sequencing (including RNA yield (and any potential DNA contamination) and DV200). Samples with DV200 below 30% were not progressed for sequencing but were included in other analyses (e.g., histopathological assessment).
Replication	We have established a unique resource to be used by the liver research community and to catalyze new discoveries in NAFLD. We present initial analyses to illustrate the utility of SteatoSITE. We used automated variable selection methods to reduce overfitting, but acknowledge that the 15-gene transcriptional risk score will require external validation if suitable cohorts can be identified
Randomization	There were no randomization procedures employed - this was a retrospective observational study. For RNA-seq analysis, principal component analysis (PCA) was performed to identify covariates that significantly correlated with the main principal components, so they could be controlled for downstream analyses. For this reason, sex was included as an additive effect in the linear model used for differential expression
Blinding	All cases were assigned a unique study ID (and the key only held by the NRS Biorepositories). Histopathological assessors and RNA-sequencing analysts were blinded to any patient information. Bioinformaticians only accessed the clinical outcome data after histopathological scoring and RNAseq analysis had been performed to enable time-to-event analysis/risk prediction etc.

Behavioural & social sciences study design

All studies must disclose on these points even when the disclosure is negative.

Study description	*Briefly describe the study type including whether data are quantitative, qualitative, or mixed-methods (e.g. qualitative cross-sectional, quantitative experimental, mixed-methods case study).*
Research sample	*State the research sample (e.g. Harvard university undergraduates, villagers in rural India) and provide relevant demographic information (e.g. age, sex) and indicate whether the sample is representative. Provide a rationale for the study sample chosen. For studies involving existing datasets, please describe the dataset and source.*
Sampling strategy	*Describe the sampling procedure (e.g. random, snowball, stratified, convenience). Describe the statistical methods that were used to predetermine sample size OR if no sample-size calculation was performed, describe how sample sizes were chosen and provide a rationale for why these sample sizes are sufficient. For qualitative data, please indicate whether data saturation was considered, and what criteria were used to decide that no further sampling was needed.*
Data collection	*Provide details about the data collection procedure, including the instruments or devices used to record the data (e.g. pen and paper, computer, eye tracker, video or audio equipment) whether anyone was present besides the participant(s) and the researcher, and whether the researcher was blind to experimental condition and/or the study hypothesis during data collection.*
Timing	*Indicate the start and stop dates of data collection. If there is a gap between collection periods, state the dates for each sample cohort.*
Data exclusions	*If no data were excluded from the analyses, state so OR if data were excluded, provide the exact number of exclusions and the rationale behind them, indicating whether exclusion criteria were pre-established.*
Non-participation	*State how many participants dropped out/declined participation and the reason(s) given OR provide response rate OR state that no participants dropped out/declined participation.*
Randomization	*If participants were not allocated into experimental groups, state so OR describe how participants were allocated to groups, and if allocation was not random, describe how covariates were controlled.*

Ecological, evolutionary & environmental sciences study design

All studies must disclose on these points even when the disclosure is negative.

Study description	*Briefly describe the study. For quantitative data include treatment factors and interactions, design structure (e.g. factorial, nested, hierarchical), nature and number of experimental units and replicates.*
Research sample	*Describe the research sample (e.g. a group of tagged Passer domesticus, all Stenocereus thurberi within Organ Pipe Cactus National Monument), and provide a rationale for the sample choice. When relevant, describe the organism taxa, source, sex, age range and any manipulations. State what population the sample is meant to represent when applicable. For studies involving existing datasets, describe the data and its source.*
Sampling strategy	*Note the sampling procedure. Describe the statistical methods that were used to predetermine sample size OR if no sample-size calculation was performed, describe how sample sizes were chosen and provide a rationale for why these sample sizes are sufficient.*
Data collection	*Describe the data collection procedure, including who recorded the data and how.*
Timing and spatial scale	*Indicate the start and stop dates of data collection, noting the frequency and periodicity of sampling and providing a rationale for*

Timing and spatial scale	*these choices. If there is a gap between collection periods, state the dates for each sample cohort. Specify the spatial scale from which the data are taken*
Data exclusions	*If no data were excluded from the analyses, state so OR if data were excluded, describe the exclusions and the rationale behind them, indicating whether exclusion criteria were pre-established.*
Reproducibility	*Describe the measures taken to verify the reproducibility of experimental findings. For each experiment, note whether any attempts to repeat the experiment failed OR state that all attempts to repeat the experiment were successful.*
Randomization	*Describe how samples/organisms/participants were allocated into groups. If allocation was not random, describe how covariates were controlled. If this is not relevant to your study, explain why.*
Blinding	*Describe the extent of blinding used during data acquisition and analysis. If blinding was not possible, describe why OR explain why blinding was not relevant to your study.*

Did the study involve field work? ☐ Yes ☐ No

Field work, collection and transport

Field conditions	*Describe the study conditions for field work, providing relevant parameters (e.g. temperature, rainfall).*
Location	*State the location of the sampling or experiment, providing relevant parameters (e.g. latitude and longitude, elevation, water depth).*
Access & import/export	*Describe the efforts you have made to access habitats and to collect and import/export your samples in a responsible manner and in compliance with local, national and international laws, noting any permits that were obtained (give the name of the issuing authority, the date of issue, and any identifying information).*
Disturbance	*Describe any disturbance caused by the study and how it was minimized.*

Reporting for specific materials, systems and methods

We require information from authors about some types of materials, experimental systems and methods used in many studies. Here, indicate whether each material, system or method listed is relevant to your study. If you are not sure if a list item applies to your research, read the appropriate section before selecting a response.

Materials & experimental systems

n/a	Involved in the study	
☐	☒	Antibodies
☐	☐	Eukaryotic cell lines
☐	☐	Palaeontology and archaeology
☐	☐	Animals and other organisms
☐	☒	Clinical data
☐	☐	Dual use research of concern
☐	☐	Plants

Methods

n/a	Involved in the study	
☐	☐	ChIP-seq
☐	☐	Flow cytometry
☐	☐	MRI-based neuroimaging

Antibodies

Antibodies used	Antibodies for MultiOmyx analysis: by staining order, were rabbit anti-TREM2 (polyclonal, ProteinTech, Catalog# 13483-1-AP, Vendor Lot ID NG) mouse anti-MNDA (253A, Abcam, Catalog# ab270556, Vendor Lot ID GR3326911), rabbit anti-CD9 (EPR2949, Abcam, Catalog# ab195422, Vendor Lot ID GR3282696), mouse anti-CD66b (G10F5, BioLegend, Catalog# 93231, ,Vendor Lot ID B276347), mouse anti-CD11B (238439, R&D Systems, Catalog# MAB16992, Vendor Lot ID KGZ0418101), rabbit anti-DC-SIGN (D7F5C, Cell Signaling Technology, Catalog# 13193, Vendor Lot ID 2), rabbit anti-Ki67 (SP6, Abcam Catalog# ab231172, Vendor Lot ID GR3277378), rabbit anti-IDO1 (SP260, Abcam Catalog# ab228468, Vendor Lot ID GR3208566), rabbit anti¬CD11c (D3V1E, Cell Signaling Catalog# 45581BF, Vendor Lot ID 2), rabbit anti-PD-L1 (SP142, Abcam Catalog# ab236238, Vendor Lot ID GR3246745), rabbit anti-CD14 (EPR3652, Abcam Catalog# ab209971, Vendor Lot ID GR316076), mouse anti-CD16 (DJ130c, ThermoFisher Scientific Catalog# MA1-84008, Vendor Lot ID TK2673378), mouse anti-CD68 (KP1, BioLegend Catalog# 98998, Vendor Lot ID B297229), mouse anti-CD163 (EDHu-1, Bio-Rad Catalog# MCA1853, Vendor Lot ID 149022A), mouse anti-HLA DQ/DR/DP (WR18, Novus Catalog# NB100-64358, Vendor Lot ID 1808), mouse anti-CD33 (44M12D3, Novus Biologicals Catalog# NBP2-22377, Vendor Lot ID 1127455612D3), mouse anti-SMA (1A4, Sigma-Aldrich Catalog# A5228, Vendor Lot ID 037M4805V).
Validation	The specificity of all antibodies was validated by board-certified pathologists employed by NeoGenomics.

Eukaryotic cell lines

Policy information about cell lines and Sex and Gender in Research

Cell line source(s)	*State the source of each cell line used and the sex of all primary cell lines and cells derived from human participants or vertebrate models.*
Authentication	*Describe the authentication procedures for each cell line used OR declare that none of the cell lines used were authenticated.*
Mycoplasma contamination	*Confirm that all cell lines tested negative for mycoplasma contamination OR describe the results of the testing for mycoplasma contamination OR declare that the cell lines were not tested for mycoplasma contamination.*
Commonly misidentified lines (See ICLAC register)	*Name any commonly misidentified cell lines used in the study and provide a rationale for their use.*

Palaeontology and Archaeology

Specimen provenance	*Provide provenance information for specimens and describe permits that were obtained for the work (including the name of the issuing authority, the date of issue, and any identifying information). Permits should encompass collection and, where applicable, export.*
Specimen deposition	*Indicate where the specimens have been deposited to permit free access by other researchers.*
Dating methods	*If new dates are provided, describe how they were obtained (e.g. collection, storage, sample pretreatment and measurement), where they were obtained (i.e. lab name), the calibration program and the protocol for quality assurance OR state that no new dates are provided.*

☐ Tick this box to confirm that the raw and calibrated dates are available in the paper or in Supplementary Information.

Ethics oversight	*Identify the organization(s) that approved or provided guidance on the study protocol, OR state that no ethical approval or guidance was required and explain why not.*

Note that full information on the approval of the study protocol must also be provided in the manuscript.

Animals and other research organisms

Policy information about studies involving animals; ARRIVE guidelines recommended for reporting animal research, and Sex and Gender in Research

Laboratory animals	*For laboratory animals, report species, strain and age OR state that the study did not involve laboratory animals.*
Wild animals	*Provide details on animals observed in or captured in the field; report species and age where possible. Describe how animals were caught and transported and what happened to captive animals after the study (if killed, explain why and describe method; if released, say where and when) OR state that the study did not involve wild animals.*
Reporting on sex	*Indicate if findings apply to only one sex; describe whether sex was considered in study design, methods used for assigning sex. Provide data disaggregated for sex where this information has been collected in the source data as appropriate; provide overall numbers in this Reporting Summary. Please state if this information has not been collected. Report sex-based analyses where performed, justify reasons for lack of sex-based analysis.*
Field-collected samples	*For laboratory work with field-collected samples, describe all relevant parameters such as housing, maintenance, temperature, photoperiod and end-of-experiment protocol OR state that the study did not involve samples collected from the field.*
Ethics oversight	*Identify the organization(s) that approved or provided guidance on the study protocol, OR state that no ethical approval or guidance was required and explain why not.*

Note that full information on the approval of the study protocol must also be provided in the manuscript.

Clinical data

Policy information about clinical studies

All manuscripts should comply with the ICMJE guidelines for publication of clinical research and a completed CONSORT checklist must be included with all submissions.

Clinical trial registration	This was not a clinical trial.
Study protocol	This was not a clinical trial. Full methodological details are provided in the manuscript.
Data collection	A total of 940 cases from the three participating NHS Scotland Biorepositories (Lothian, Greater Glasgow & Clyde, and Grampian) were included, representing the full histological spectrum from normal liver tissue to NAFLD-related cirrhosis. Cases with a liver

	tissue sample acquired between January 2000 and October 2019 were selected. All patients were years of age at the tissue sampling date. Data from Electronic Health Records and national datasets were retrieved, where available, from a period between ten years before the tissue sampling date until May 2020.
Outcomes	We collected all relevant clinical outcomes according to recent expert consensus guidelines for using administrative coding in Electronic Health Record-based research of NAFLD.

Dual use research of concern

Policy information about dual use research of concern

Hazards

Could the accidental, deliberate or reckless misuse of agents or technologies generated in the work, or the application of information presented in the manuscript, pose a threat to:

No	Yes	
☒	☐	Public health
☒	☐	National security
☒	☐	Crops and/or livestock
☒	☐	Ecosystems
☒	☐	Any other significant area

Experiments of concern

Does the work involve any of these experiments of concern:

No	Yes	
☒	☐	Demonstrate how to render a vaccine ineffective
☒	☐	Confer resistance to therapeutically useful antibiotics or antiviral agents
☒	☐	Enhance the virulence of a pathogen or render a nonpathogen virulent
☒	☐	Increase transmissibility of a pathogen
☒	☐	Alter the host range of a pathogen
☒	☐	Enable evasion of diagnostic/detection modalities
☒	☐	Enable the weaponization of a biological agent or toxin
☒	☐	Any other potentially harmful combination of experiments and agents

Plants

Seed stocks	*Report on the source of all seed stocks or other plant material used. If applicable, state the seed stock centre and catalogue number. If plant specimens were collected from the field, describe the collection location, date and sampling procedures.*
Novel plant genotypes	*Describe the methods by which all novel plant genotypes were produced. This includes those generated by transgenic approaches, gene editing, chemical/radiation-based mutagenesis and hybridization. For transgenic lines, describe the transformation method, the number of independent lines analyzed and the generation upon which experiments were performed. For gene-edited lines, describe the editor used, the endogenous sequence targeted for editing, the targeting guide RNA sequence (if applicable) and how the editor was applied.*
Authentication	*Describe any authentication procedures for each seed stock used or novel genotype generated. Describe any experiments used to assess the effect of a mutation and, where applicable, how potential secondary effects (e.g. second site T-DNA insertions, mosiacism, off-target gene editing) were examined.*

ChIP-seq

Data deposition

☐ Confirm that both raw and final processed data have been deposited in a public database such as GEO.

☐ Confirm that you have deposited or provided access to graph files (e.g. BED files) for the called peaks.

Data access links<br>*May remain private before publication.*	*For "Initial submission" or "Revised version" documents, provide reviewer access links. For your "Final submission" document, provide a link to the deposited data.*
Files in database submission	*Provide a list of all files available in the database submission.*

Genome browser session
(e.g. UCSC)

Provide a link to an anonymized genome browser session for "Initial submission" and "Revised version" documents only, to enable peer review. Write "no longer applicable" for "Final submission" documents.

Methodology

Replicates

Describe the experimental replicates, specifying number, type and replicate agreement.

Sequencing depth

Describe the sequencing depth for each experiment, providing the total number of reads, uniquely mapped reads, length of reads and whether they were paired- or single-end.

Antibodies

Describe the antibodies used for the ChIP-seq experiments; as applicable, provide supplier name, catalog number, clone name, and lot number.

Peak calling parameters

Specify the command line program and parameters used for read mapping and peak calling, including the ChIP, control and index files used.

Data quality

Describe the methods used to ensure data quality in full detail, including how many peaks are at FDR 5% and above 5-fold enrichment.

Software

Describe the software used to collect and analyze the ChIP-seq data. For custom code that has been deposited into a community repository, provide accession details.

Flow Cytometry

Plots

Confirm that:

- [ ] The axis labels state the marker and fluorochrome used (e.g. CD4-FITC).
- [ ] The axis scales are clearly visible. Include numbers along axes only for bottom left plot of group (a 'group' is an analysis of identical markers).
- [ ] All plots are contour plots with outliers or pseudocolor plots.
- [ ] A numerical value for number of cells or percentage (with statistics) is provided.

Methodology

Sample preparation

Describe the sample preparation, detailing the biological source of the cells and any tissue processing steps used.

Instrument

Identify the instrument used for data collection, specifying make and model number.

Software

Describe the software used to collect and analyze the flow cytometry data. For custom code that has been deposited into a community repository, provide accession details.

Cell population abundance

Describe the abundance of the relevant cell populations within post-sort fractions, providing details on the purity of the samples and how it was determined.

Gating strategy

Describe the gating strategy used for all relevant experiments, specifying the preliminary FSC/SSC gates of the starting cell population, indicating where boundaries between "positive" and "negative" staining cell populations are defined.

- [ ] Tick this box to confirm that a figure exemplifying the gating strategy is provided in the Supplementary Information.

Magnetic resonance imaging

Experimental design

Design type

Indicate task or resting state; event-related or block design.

Design specifications

Specify the number of blocks, trials or experimental units per session and/or subject, and specify the length of each trial or block (if trials are blocked) and interval between trials.

Behavioral performance measures

State number and/or type of variables recorded (e.g. correct button press, response time) and what statistics were used to establish that the subjects were performing the task as expected (e.g. mean, range, and/or standard deviation across subjects).

Acquisition

Imaging type(s) — *Specify: functional, structural, diffusion, perfusion.*

Field strength — *Specify in Tesla*

Sequence & imaging parameters — *Specify the pulse sequence type (gradient echo, spin echo, etc.), imaging type (EPI, spiral, etc.), field of view, matrix size, slice thickness, orientation and TE/TR/flip angle.*

Area of acquisition — *State whether a whole brain scan was used OR define the area of acquisition, describing how the region was determined.*

Diffusion MRI ☐ Used ☐ Not used

Preprocessing

Preprocessing software — *Provide detail on software version and revision number and on specific parameters (model/functions, brain extraction, segmentation, smoothing kernel size, etc.).*

Normalization — *If data were normalized/standardized, describe the approach(es): specify linear or non-linear and define image types used for transformation OR indicate that data were not normalized and explain rationale for lack of normalization.*

Normalization template — *Describe the template used for normalization/transformation, specifying subject space or group standardized space (e.g. original Talairach, MNI305, ICBM152) OR indicate that the data were not normalized.*

Noise and artifact removal — *Describe your procedure(s) for artifact and structured noise removal, specifying motion parameters, tissue signals and physiological signals (heart rate, respiration).*

Volume censoring — *Define your software and/or method and criteria for volume censoring, and state the extent of such censoring.*

Statistical modeling & inference

Model type and settings — *Specify type (mass univariate, multivariate, RSA, predictive, etc.) and describe essential details of the model at the first and second levels (e.g. fixed, random or mixed effects; drift or auto-correlation).*

Effect(s) tested — *Define precise effect in terms of the task or stimulus conditions instead of psychological concepts and indicate whether ANOVA or factorial designs were used.*

Specify type of analysis: ☐ Whole brain ☐ ROI-based ☐ Both

Statistic type for inference (See Eklund et al. 2016) — *Specify voxel-wise or cluster-wise and report all relevant parameters for cluster-wise methods.*

Correction — *Describe the type of correction and how it is obtained for multiple comparisons (e.g. FWE, FDR, permutation or Monte Carlo).*

Models & analysis

n/a	Involved in the study
☐	☐ Functional and/or effective connectivity
☐	☐ Graph analysis
☐	☐ Multivariate modeling or predictive analysis

Functional and/or effective connectivity — *Report the measures of dependence used and the model details (e.g. Pearson correlation, partial correlation, mutual information).*

Graph analysis — *Report the dependent variable and connectivity measure, specifying weighted graph or binarized graph, subject- or group-level, and the global and/or node summaries used (e.g. clustering coefficient, efficiency, etc.).*

Multivariate modeling and predictive analysis — *Specify independent variables, features extraction and dimension reduction, model, training and evaluation metrics.*
